# Time-based tracking of temperature and humidity of emergency medical service rapid response vehicles in Qatar: a prospective observational study

**DOI:** 10.1186/s12873-025-01255-3

**Published:** 2025-07-01

**Authors:** Nour Alhuda Alaghawani, Abrar Mohamed, Ahmed Makhlouf, Habib Kerkeni, Loua Al Shaikh, Guillaume Alinier, Alaaldin M. Alkilany, Ousama Rachid

**Affiliations:** 1 College of Pharmacy, QU Health, Qatar University, Doha, Qatar; 2 Hamad Medical Corporation Ambulance Service, Doha, Qatar; 3 Weill Cornell Medicine-Qatar, Doha, Qatar; 4 School of Health, Medicine, and Life Sciences, University of Hertfordshire, Hatfield, UK; 5 Faculty of Health and Life Sciences, Northumbria University, Newcastle Upon Tyne, UK

**Keywords:** Temperature, Humidity, Mean kinetic temperature, Emergency medical services, Ambulances, Rapid response cars

## Abstract

**Background:**

Paramedics working in emergency medical services (EMS) routinely administer life-saving medications to patients under urgent conditions. However, these medications are frequently subjected to undocumented fluctuations in environmental conditions, particularly temperature and humidity, which may lead to drug degradation and potentially compromise patient safety. In countries like the State of Qatar, known for its elevated temperatures and humidity, the environmental exposure of EMS medications stored in rapid response cars (RRCs) has not yet been systematically assessed. In this study, we aimed to evaluate the fluctuations in temperature and humidity experienced by ambulatory medications over a 12-month period.

**Methods:**

Six RRCs, each with three temperature and humidity loggers were utilized to collect real-life environmental data. Two loggers were placed in two paramedic bags stored at the back of the RRCs while a third logger was attached in the middle inside each car. Temperature and humidity readings were recorded at 10-minute intervals over 12 months for assessment. Data was then extracted using ElitechLog software, visualized using Python, and statistically analyzed. The mean kinetic temperature (MKT) was also calculated.

**Results:**

Temperature values reached 59.1 and 65.7 °C in the bags and inside the RRC, respectively. The MKT exceeded the United States Pharmacopeia recommendations of 30 °C, and in some instances, it exceeded 50 °C. Little to no difference was observed between the two bags in each car however, greater temperature values and MKT violations were reported by the centrally located sensor inside the RRCs.

**Conclusion:**

The reported MKT violations highlight the need to develop and implement improved storage strategies for EMS medications in emergency vehicles operating in extremely hot climates. However, given that medication bags are frequently exposed to high ambient temperatures when carried outside by paramedics, storage solutions alone are insufficient to fully prevent deviations from manufacturers’ recommended conditions. This underscores the importance of specialized training for paramedics on stringent medication handling protocols to minimize temperature exposure and ensure drug safety and efficacy.

**Clinical trial number:**

Not applicable.

**Supplementary Information:**

The online version contains supplementary material available at 10.1186/s12873-025-01255-3.

## Introduction

In the State of Qatar, the Hamad Medical Corporation Ambulance Service (HMCAS) receives over 250,000 calls annually for cases requiring both emergency and non-emergency medical services [1–3]. While working on these cases, paramedics are often required to provide medical interventions using life-saving medications carried in their paramedic bags, allowing the medications to routinely get exposed to harsh environmental conditions such as heat, humidity, light, and agitation. The HMCAS paramedic bags mostly carry liquid dosage forms such as those packaged in ampoules or vials, which are generally more sensitive to environmental conditions and have more stringent storage requirements compared to solid dosage forms like tablets or capsules (Appendix 1). To maintain their stability, optimum storage conditions as per the manufacturer label must be adhered to. This ensures their overall integrity and effectiveness, prevents harm to the patient, and reduces the overall health care costs [4].

In Qatar, the maximum temperature and relative humidity (RH) readings recorded across 12 months were 50 °C and 85%, respectively [5], signifying the relatively extreme environment under which drug stability needs to be assessed. The World Health Organization (WHO) has classified Qatar to be under climate zone IVa (hot humid) for stability testing [6]. Guidelines set by the International Council for Harmonisation of Technical Requirements for Pharmaceuticals for Human Use (ICH), WHO, and European Medicine Agency (EMA) suggested a maximum temperature of 30 °C and a maximum RH of 65% for long term medication storage in hot and humid regions (Table 1) [6–8]. The United States Pharmacopeia (USP) on the other hand, suggested that medications are to be stored in dry areas (with average RH < 40%) that are protected from excessive heat (temperatures > 40 °C), and freezing temperatures, unless stated otherwise on the label [9].

**Table 1:** Guideline recommendations on maximum temperature and humidity long term storage in hot and humid regions

Guideline	Temperature (°C)	Relative humidity (%)
International Council for Harmonisation (ICH)	30	65
World Health Organization (WHO)	30	65
European Medicine Agency (EMA)	30	65
United States Pharmacopeia (USP)	30	40

In the literature, there is a significant gap in reporting temperature and humidity levels in out-of-hospital medical settings, especially in the Middle East and North Africa (MENA) climate zone IV region. Few studies reported on other milder climate zones. For instance, temperatures were found to exceed the recommended 25 °C in a six week study performed in four EMS locations in South Africa, where sensors placed in emergency cars and medication facilities over summer recorded the temperature every 15 min [10]. Several other studies conducted in either animal veterinary cars, EMS advanced life support drug bags, EMS helicopters, or even under different storage conditions (such as a refrigerator, at room temperature, and in an emergency physician transport car) concluded similar outcomes of potential safety concerns on medication storage, highlighting that even at regular room temperatures, lifesaving medications could easily degrade and lose stability, thus stressing the significance of environmental temperature and humidity monitoring in EMS drug storage areas [11–19]. Using medication bags or boxes alone to store medications in cars was also reported as unsafe, especially during the summer months, even when placed in garage-like environments [20].

**Fig. 1:**
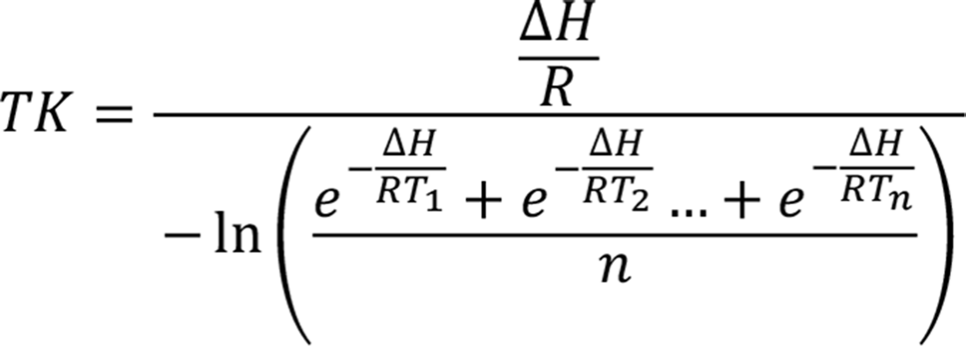
Mean kinetic temperature (MKT) Eq. [22]. TK is the mean kinetic temperature in Kelvin; ΔH is the activation energy; R is the gas constant; T1-Tn are the temperatures at each point in Kelvin; n is the number of temperature sample points

Taken together, there is limited reporting on temperature and humidity during medication storage in EMS settings of zone IV climates [21]. Reports in other zones covered short durations, making it difficult to evaluate seasonal climate fluctuations over a full calendar year. Above all, most missed to report the mean kinetic temperature (MKT) value (Fig. 1), which is a “single calculated temperature at which the total amount of degradation over a particular period is equal to the sum of the individual degradations that would occur at various temperatures” according to the USP [22]. MKT is essential when determining drug degradation based on storage conditions, as it shows the effect of temperature fluctuations on a product over time, thus making it a vital component in assessing the role of temperature excursions on drug degradation during storage [21, 23, 24]. As current EMS services in Qatar do not employ frequent monitoring of storage conditions in their vehicles, the lifesaving medications which they carry are constantly subjected to unknown environmental conditions raising a question on their stability and integrity. Thus, this study aimed to map the unknown storage conditions including temperature and humidity as well as excursions in MKT within HMCAS rapid response cars (RRCs), to assess the need for improved drug storage conditions that would ensure medication stability and ultimately patient safety. By documenting the extent of environmental excursions and their potential impact on drug stability and efficacy, our study provides a foundation for policy reforms that could enhance not only local EMS practices but also those in regions with similar climatic challenges.

## Methods

### Study design and setting

**Fig. 2:**
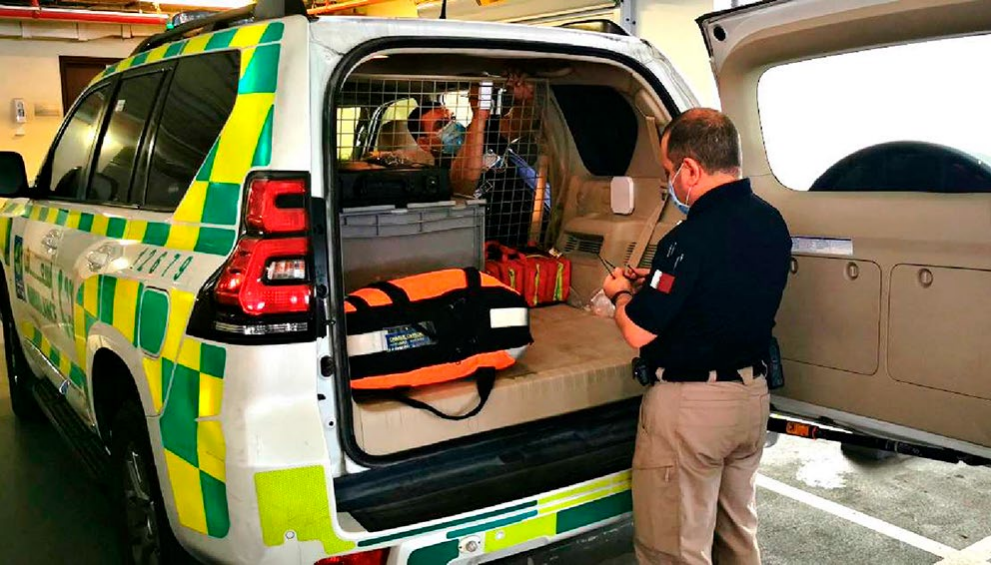
A hamad medical corporation ambulance service rapid response car utilized in this study

This prospective observational study was carried out under real-world conditions and similar to other studies focused on out-of-hospital settings [10, 11, 13–19, 21, 25–30]. The study spanned from January 2021 through January 2022, and it involved six HMCAS Charlie units [RRC with a Critical Care Paramedic and Critical Care Assistant [31], each with three temperature/humidity data loggers (Fig. 2). These RRCs were not confined to one geographical location as throughout their shifts, they may have travelled to any location across Qatar; however, they had to return to their starting point at the end of the shift. Given the relatively small size of Qatar, and the consistent return to a home base, the impact of geographical variation on data logger recordings was expected to be minimal.

Permission was granted by the HMCAS Research Oversight Committee and Hamad Medical Corporation Medical Research Centre (MRC-01-20-411) to conduct the study. It was considered as exempt from ethical approved as it was classified as non-human subject research.

### Data collection

**Fig. 3:**
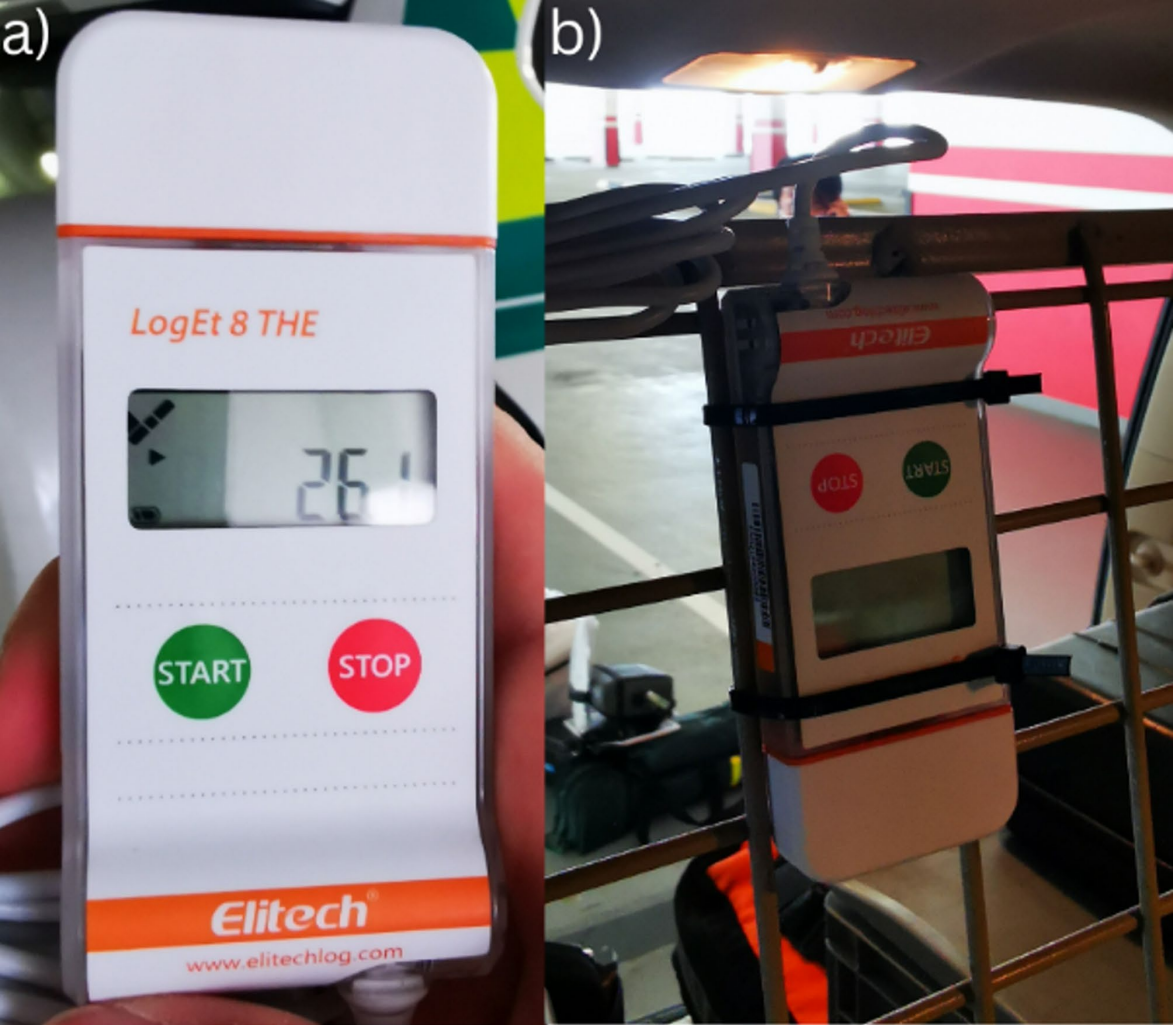
**a**) LogEt 8 THE data logger, **b**) Data logger in the centre of the rapid response car (attached to the boot separator above the back row of seats)

Data collection was done using Elitech LogEt 8 THE data loggers (Fig. 3a), which had a temperature and humidity measuring range of -40 to 85 °C and 10 to 90%, respectively (Elitech UK LTD). The logging interval was set so that measurements were made every 10 min, and the overall memory span of the loggers was up to 16,000 temperature and humidity points each. The loggers were pre-certified by the manufacturing company to ensure the reliability of the measurements. After the logger’s memory got filled, the data was exported for use as an Elitech file compatible with the ElitechLogWin V7.0.0 Beta 15 software program.

**Fig. 4:**
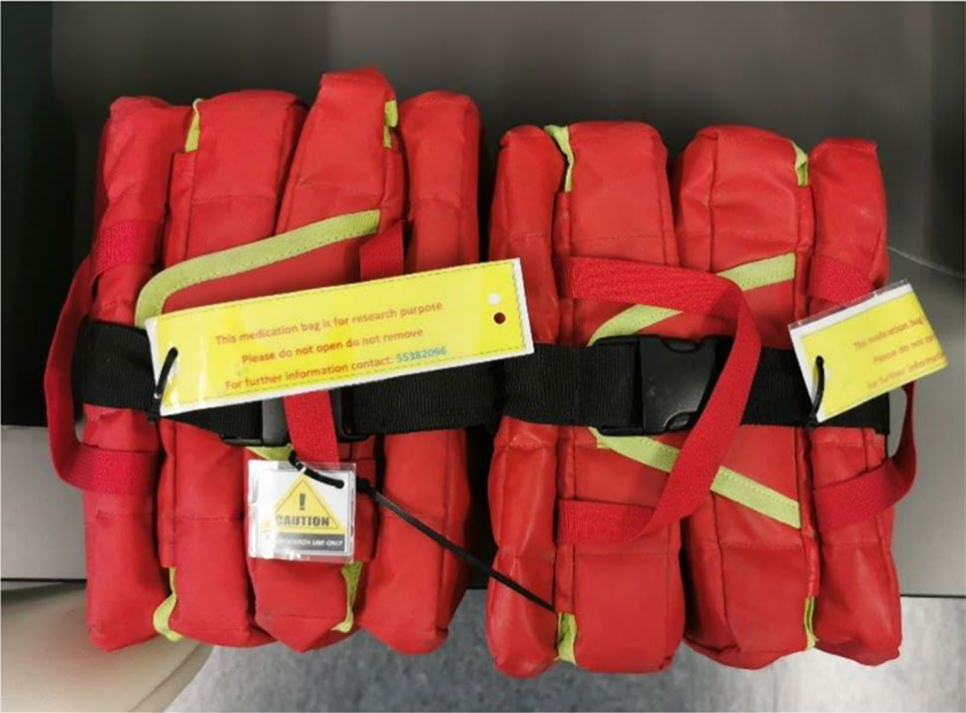
Paramedic bags used as they were zip tied with the labels attached specifying these were for research purpose only and not to be removed

One data logger was fixed inside, on the metal mesh in the middle of the RRC (Fig. 3b) to record the environmental conditions inside the car itself compared to the conditions inside the insulated medication storage bags to assess their effectiveness in minimizing temperature/humidity fluctuations [32]. To avoid disrupting paramedics duties, we utilized dummy research medication bags for data collection, which were identical to the operational paramedic bags typically filled to capacity, leaving no room to accommodate extra research items such as data loggers. Therefore, two of the three data loggers used were each placed in two research medication bags that were zip tied together by the handles, with a label indicating that they were for research purposes (Fig. 4). Just like the operational paramedic bags, they contained similar content which the paramedics would regularly use on their shift. Unlike the other studies, this one utilized two bags placed in the same environment to validate the final results as they would be expected to be the same.

New data loggers were also placed in each RRC every three months to ensure continuous monitoring, as that is roughly the duration it took for ~ 16,000 data points to be collected. Therefore, four loggers were placed over 12 months in each of the three locations of the six cars, giving us 72 exported Elitech files with approximately 1.15 million data points in total.

### Data and statistical analysis

Preliminary Python graphs were generated from the collected data. These initial graphs were utilized for general data overview and visual diagnosis. These graphs were then assessed for any abnormalities such as missing or overlapping data. Then, the 72 files of raw data were exported to Microsoft Excel, where the date and time of the missing data and other anomalies were noted. Finally, four Excel sheets covering the four periods from each location in the car were compiled into one yearly sheet, which underwent computational and statistical analysis using Python version 3.9 to automate the process of reading, processing, and visualizing the data from Microsoft Excel.

The plots’ error bars (mean ± SD) are based on daily average temperatures, not the raw 10-minute readings. Each daily average is computed from 144 measurements, and by the Central Limit Theorem such daily means are approximately normally distributed even if the underlying measurements are skewed [33, 34]. Not only was daily averaging calculated, but we even made further aggregation of daily averages into monthly averages which even magnifies the central tendency towards normal distribution behavior. In other words, the distribution of daily averages is near-normal, and the skewness is greatly reduced through averaging. For our formal comparisons between the two loggers in each pair of bags, we employed a non-parametric Mann–Whitney U test, which does not assume normality, to account for the skewed nature of the raw data. Using mean ± SD for illustrating the daily average temperatures provides a clear depiction of day-to-day variability, while using non-parametric tests ensured that no invalid normality assumptions influenced the comparative results.

The recommended upper specification limits (USL) of 30 °C for temperature, 65% for humidity, and 30 °C for MKT were indicated on the graphs. The average daily MKT for each month and the number of MKT violations were also plotted. Python was used to calculate the averages and coefficients of variation for each bag and for the in-car readings within each RRC. A p-value of < 0.05 was considered statistically significant.

## Results

Throughout the study period, the minimum/maximum temperatures and RH observed in both paramedic bags and in the middle inside the six cars were 12.2/59.1 °C and 9.4/65.7 °C for temperature, and 14.1/98.4% and 11.7/99.0% for RH, respectively (Table 2). The percentage of data crossing the temperature of 30 °C and RH of 65% recommended by several guidelines was examined (Appendix 2). The logger in the middle of RRC 2 experienced the largest deviations from the suggested temperature with 39.1% of the data crossing 30 °C. The largest percentage of RH deviations were observed in the middle of RRC 4 with 1.7% of the data being beyond the recommended RH.

**Table 2:** Maximum, minimum, and mean temperature and humidity from the combined bags and inside the vehicle

Rapid Response Car Number	**Bags 1 and 2 Combined**						Inside in the middle of the Rapid Response Car					
	Temperature (°C)			Relative Humidity (%)			Temperature (°C)			Relative Humidity (%)		
	Min	Max	Mean (n)	Min	Max	Mean (n)	Min	Max	Mean (n)	Min	Max	Mean (n)
1	14.9	43.6	27.4 (102,056)	15.2	78.7	41.5 (102,056)	10.7	59.9	28.1 (50,311)	15.3	96.4	42.7 (50,311)
2	13.6	59.1	28.3 (106,563)	14.1	97.6	39.1 (106,563)	9.40	64.6	28.4 (48,862)	12.4	95.4	40.6 (48,862)
3	13.2	50.7	27.7 (106,562)	16.2	96.5	41.6 (106,562)	13.6	58.8	25.2 (32,102)	15.1	95.0	42.9 (32,102)
4	13.8	57.7	28.2 (105,410)	16.7	93.1	40.7 (105,410)	12.4	65.7	27.8 (52,705)	12.1	96.8	42.3 (52,705)
5	12.2	57.0	27.1 (107,714)	15.7	98.4	40.2 (107,714)	10.5	65.7	26.0 (46,209)	12.1	99.0	41.9 (46,209)
6	14.2	46.9	27.4 (94,788)	17.9	76.8	43.1 (94,788)	11.9	57.7	26.6 (29,014)	11.7	88.5	42.1 (29,014)

The overall mean temperature and RH across all bags and in the middle of the RRCs in the final 18 data sets (three data loggers in each of the six RRCs: 3 × 6 = 18) was 27.5 °C and 41.2%; while separately, the mean climate parameters in the bags were 27.7 °C and 41%, and in the middle of the RRCs they were 27.2 °C and 42%.

Similar temperature means were exhibited in the bags which ranged from 26.9 to 28.5 °C. For RH, the bags had a mean that ranged from 39.0 to 44.1%. Similarly, in the middle of the RRCs, the mean temperature ranged from 26.0 °C to 28.4 °C, while the mean RH ranged from 40.6 to 42.9%. There is no significant difference in the data between bag 1 and bag 2 of each RRC (Table 3).

**Table 3:** Coefficient of variance (CV) for each bag, the CV difference, CV difference %, the Mann-Whitney U test, and the *p*-value

	CV for bag 1	CV for bag 2	CV Difference	CV Difference %	Mann- Whitney	p-Value
**RRC 1 bag 1** **vs bag 2**	16.577642	15.640378	0.937264	5.82	60349	0.343
**RRC 2 bag 1** **vs bag 2**	19.049145	18.332302	0.716843	3.84	69927	0.369
**RRC 3 bag 1** **vs bag 2**	18.757318	18.471377	0.285941	1.54	65016	0.418
**RRC 4 bag 1** **vs bag 2**	18.597629	18.959705	0.362076	1.93	67617	0.925
**RRC 5 bag 1** **vs bag 2**	14.360091	14.682630	0.322539	2.22	71253	0.174
**RRC 6 bag 1** **vs bag 2**	16.663998	16.899134	0.235136	1.40	57119	0.222

### Temperature

The temperature data obtained from the loggers was first plotted as a yearly aggregate to show fluctuations across the whole year (Appendix 3). Standard temperature fluctuations were observed throughout the graphs with a gradual increase during summer and decrease during winter. Several peaks and troughs were deviating from the standard trend. For instance, both bags in RRC 1 (Appendix 3a and b) showed a decrease in reported values with temperatures remaining below 25 °C from the 4th of September till the 4th of October when the new logger was first added. Other out of range temperature peaks were also seen such as in January 2021 within bags 1 and 2 of RRC 6 (Appendix 3p and q) when temperatures over 30 °C were observed. Missing data can also be noted in the graphs such as the period between 24th of July 2021 and 4th of January 2022 inside RRC 6 (Appendix 3r). Possible reasons leading to missing data have been identified including accidental manual stopping of the logger and reaching maximum capacity of the logger memory, as indicated in the graphs.

### Humidity

Similarly, the data obtained for the RH was graphed as a yearly aggregate showing all the fluctuations across the year (Appendix 4). It can be observed in most graphs that the RH did not fluctuate much beyond the recommendations. The average RH recorded in the combined bags and inside the RRCs ranged between 39.1% and 43.1%, which is within the recommended guideline limit of 65% (Table 2). The maximum observed RH was 98.4% in the bags and 99.0% inside the RRCs. The average percentage of data exceeding the recommended limit was around 1.1% from each data logger storage location (Appendix 2). Therefore, the RH readings were reported only, without further data analysis.

### Mean kinetic temperature

**Fig. 5:**
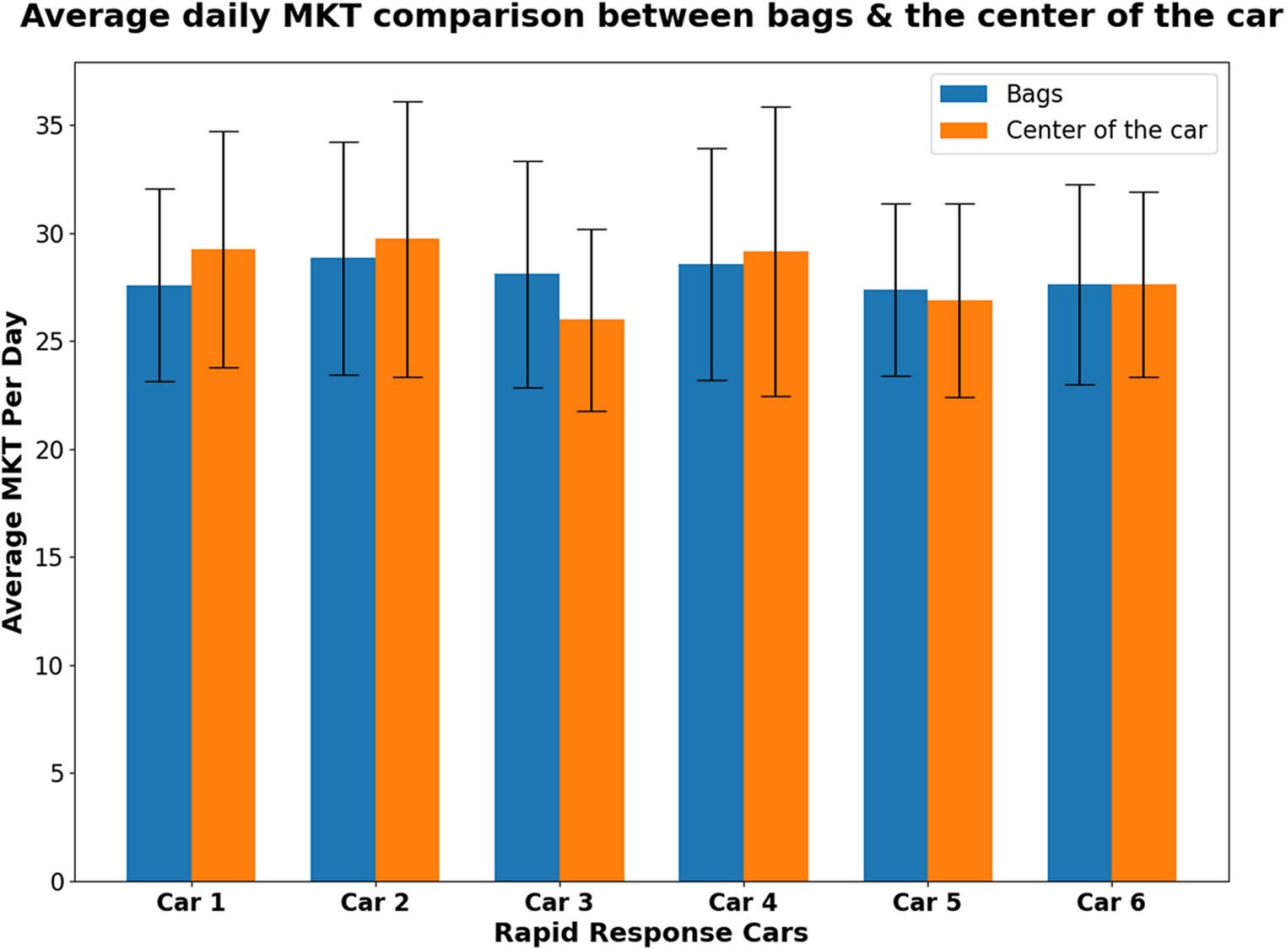
The average daily mean kinetic temperature (MKT) values over 12 months in the bags and inside the rapid response cars

**Fig. 6:**
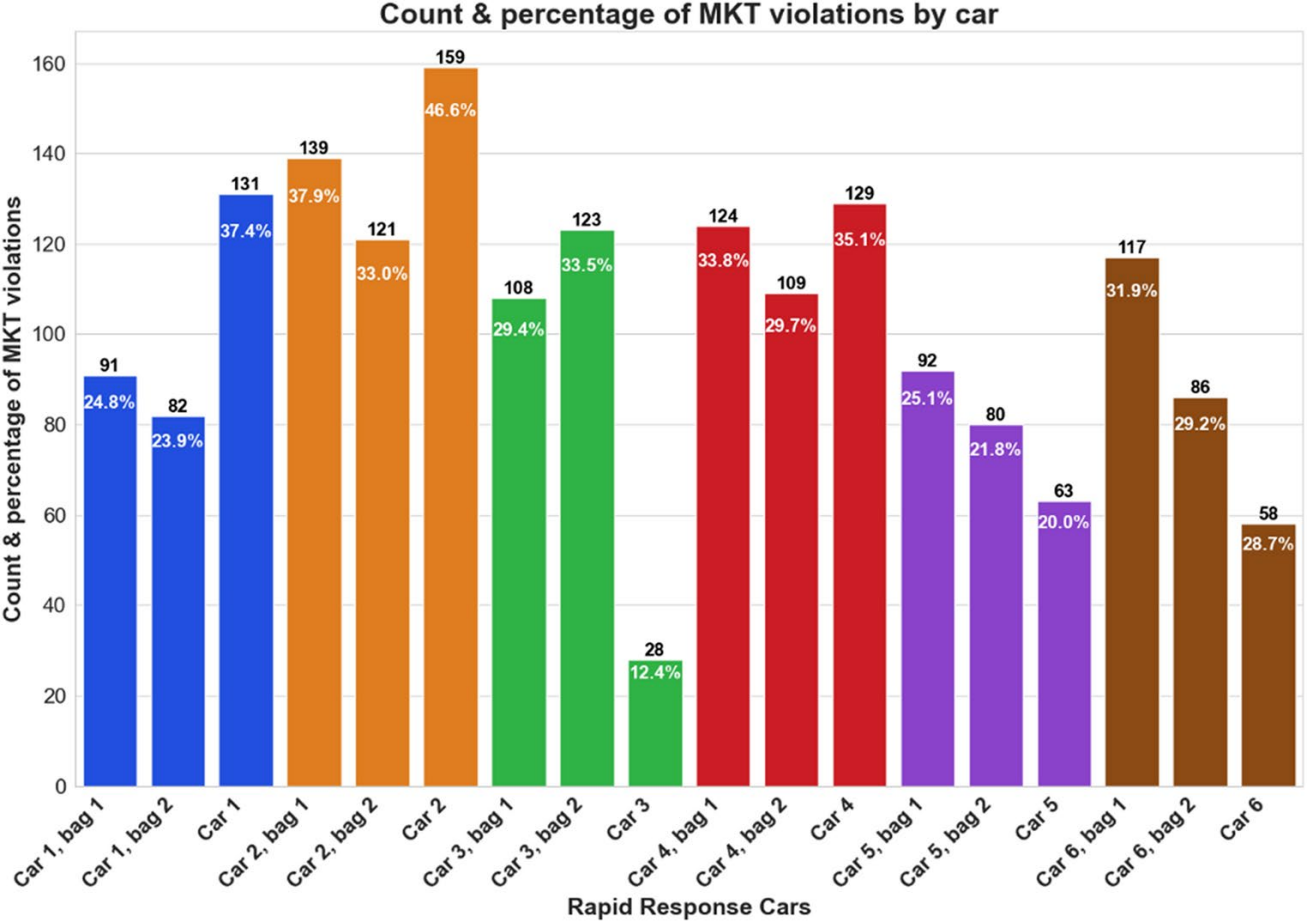
Count (above bar) and percentage (inside bar) of daily mean kinetic temperature (MKT) violations (above 30 °C) over 12 months by data logger storage location. The bars are color-coded based on their attributable rapid response car

**Fig. 7:**
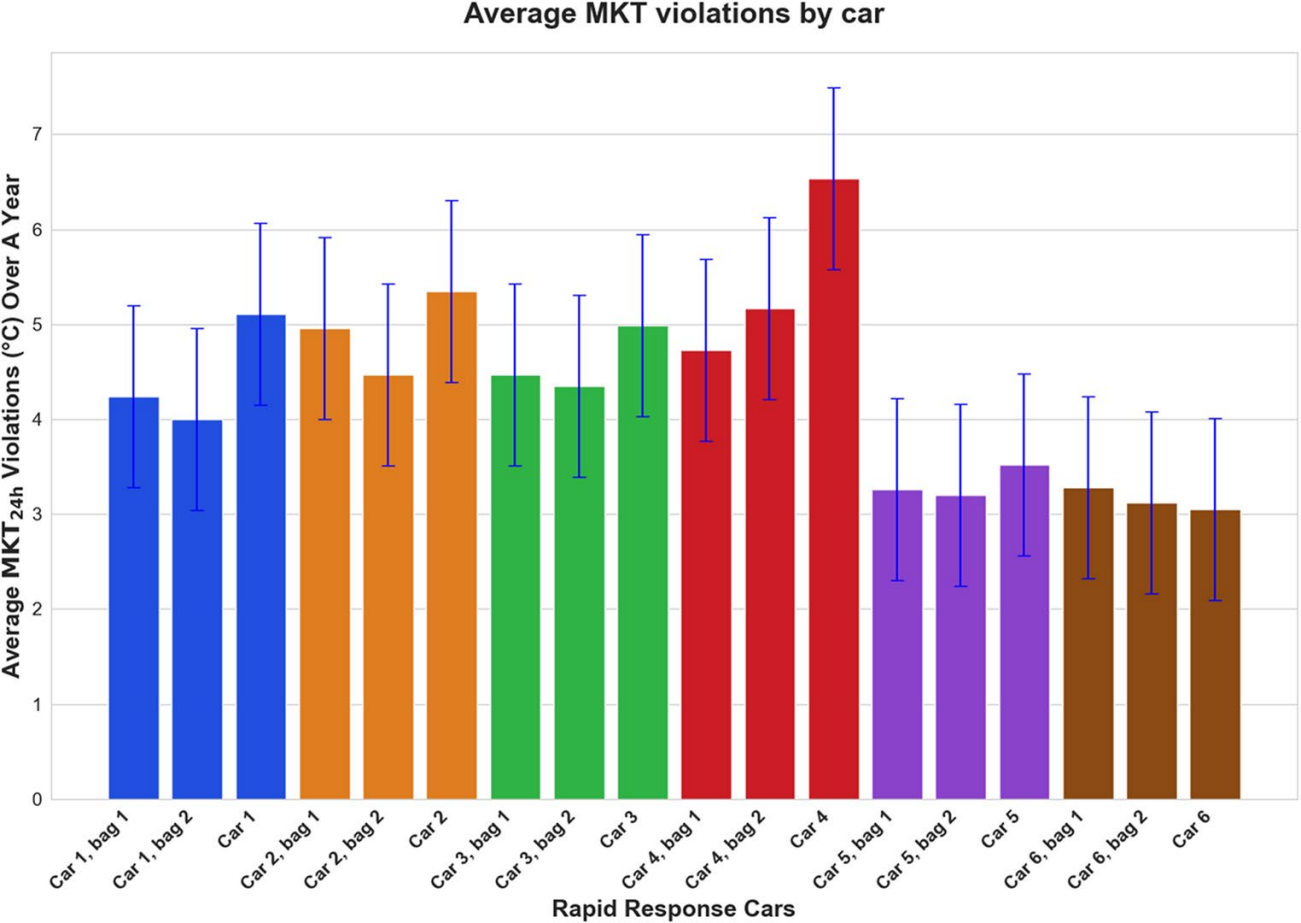
Average value (difference to 30 °C) of daily mean kinetic temperature (MKT) violations over 12 months by data logger storage location. The bars are color-coded based on their attributable rapid response car

From May through October, the average monthly MKT per day for the bags and inside the RRCs, exceeded the USL (Appendices 5–8). The average daily MKT was greater inside the RRC than in the two bags combined in RRC 1,2, and 4; however, 3,5, and 6 showed slight deviations (Fig. 5). Figure 6 shows the frequency and percentage of violations observed (MKT values exceeding 30 °C) by data logger location, out of the total number of calculated MKTs. In most cases the MKT violations inside the RRCs were greater than those observed in the bags. Figure 7 represents the MKT violations observed (MKT value difference to 30 °C). The average MKT violations inside the RRCs were greater than those of the bags. RRC 6 showed significant loss of data in comparison to other RRCs. The figures’ error bars (mean ± SD) are based on daily average temperatures, not the raw 10-minute readings.

## Discussion

Temperatures in Qatar are typically elevated compared to other countries. As out-of-hospital environments are usually uncontrolled, especially temperature [35], we aimed to map the conditions in an ambulatory setting to assess the need for improved storage conditions that would ensure medication stability and patient safety. Throughout the study period (January 2021 until January 2022), the maximum temperatures observed in the insulated bags and inside the RRCs were higher than previous reports of the maximum reported temperatures of 50 °C in open air spaces in Qatar [5, 36], and exceeded guideline recommendations. The minimum temperatures detected inside the bags and inside the RRCs in this study were consistent with a previous study that reported minimal temperatures of 11 °C [36]. The mean temperature of the bags and inside the RRCs fell in line with previously reported average temperatures in Qatar [36]. Considering that mean temperatures are calculated on a yearly basis and the averages are relatively close to the recommendations, this signifies that temperatures reported might be normalized when calculated over a prolonged period.

Around 30–40% of our recorded data surpassed the recommended guideline temperature of 30 °C which denoted a potential concern regarding the stability of medications carried in EMS cars (Appendix 2). According to the USP, temperature excursions beyond 40 °C increase the likelihood of drugs to degrade [23], which highlights that the issue of uncontrolled medication storage in EMS vehicles remains persistent, stressing the need to routinely assess current storage conditions in ambulances and other medical response vehicles. Most of the medications utilized in this study have storage recommendations of 20–25 °C (room temperature), with instructions to avoid light, extreme temperatures, and even recommending refrigeration for some (Appendix 1). Thus, the high temperatures and MKT excursions observed in this study highlight possible effect on drug stability, which can directly influence the effectiveness of these life-saving medications. Drugs which have been reported to experience degradation may have experienced reduced efficacy, which could be life-threatening in an emergency [13, 19, 26, 27].

In addition to environmental exposure, the frequency with which medications are used and restocked also plays a role in potential degradation risk. Monthly consumption data from HMCAS show a wide range of usage frequencies (Appendix 1). For instance, salbutamol nebules and adrenaline ampoules were used frequently (average monthly use of 625 and 375 units, respectively), suggesting rapid turnover and reduced cumulative exposure. Conversely, drugs such as glyceryl trinitrate (63 units/month) and salbutamol (4 units/month) ampoules were used much less frequently, implying that they may remain in the bags for extended periods. These low-rotation medications are at greater risk of prolonged environmental stress, highlighting the need for tailored monitoring and possibly shorter shelf-life cycles for certain items in EMS storage.

### Temperature

The 10-minute interval temperature readings over 12 months from the bags and inside the car (Appendix 3) reported from each RRC show a general and expected seasonal trend with higher temperatures during Qatar’s summer season and lower temperatures during the winter season. Missing data was observed, namely in RRC 6 bag 2, and inside of RRC 2, 3, 4, 5, and 6 (Appendix 3f,i,l,o,q,r), which is likely attributed to the logger’s memory reaching maximum capacity or due to it being accidentally turned off as bags and other pieces of equipment were stored together at the back of the RRC.

Peaks or troughs which surpassed the normal range of other points within the same timeframe were considered abnormal. Logistically, the cars on shift had their air conditioning operating for almost 90% of the time with the air flow facing the study bags which may have caused a sudden drop in temperatures especially during summer. Additionally, the bags were at the back of the car and were sometimes covered by several pieces of equipment used by paramedics, which may have reduced ventilation leading to higher temperatures. Furthermore, the tinted windows of the car and the thermo-insulated paramedic bags used may have reduced temperatures. By using two data loggers in two adjacent bags, we were able to confirm minimal variability in the temperature records observed between both bags of each RRC, especially RRCs 3 and 6 (Table 3), as it helped prove the expected consistency between both bags, validating the results and showing reliability of the data loggers’ measurements.

The loggers placed in the middle of the car were directly exposed to extreme and rapid temperature fluctuations, such as those occurring when the car doors were opened and closed, with direct sunlight exposure, and conduction from the metal net on which they were attached. This can be observed in the recordings of temperatures inside the RRCs, where the maximum and minimum temperatures observed were higher and lower than those from the bags, respectively.

In addition to absolute temperature levels, the duration and variability of exposure are key factors in medication degradation. While most guidelines (e.g., ICH, USP) define thresholds such as 30 °C for long-term storage, they do not always specify how long a short-term excursion above this threshold can be tolerated before degradation becomes clinically significant. Some thermolabile drugs may begin to degrade after even brief high-temperature exposures, while others may tolerate excursions up to 40 °C for limited time windows. Furthermore, the daily fluctuation or “cycling” of temperature and humidity, where values rise and fall repeatedly, can accelerate degradation processes more than constant conditions, particularly due to stress on packaging and excipients. This reinforces the importance of using MKT in our study, as it accounts for both the magnitude and frequency of these fluctuations and provides a better representation of cumulative thermal stress. Future studies should aim to investigate not just the presence of temperature excursions, but also the degradation profiles of specific drugs in response to time-based, fluctuating exposure patterns like those observed in EMS environments.

### Mean kinetic temperature

In our study, we calculated daily MKT values (Appendix 5), compared to previous studies that reported MKT on a weekly and/or monthly basis [13, 25, 37]. These long-term calculations may mask daily MKT excursions [23, 38]. To ease analysis, we have reported the monthly MKT averages for bags and RRCs combined, two bags combined alone, and RRC alone (Appendix 6–8). Several MKT values exceeded 50 °C (Appendix 5f), particularly those reported from May through October (Appendix 6–8, e-j) exceeding the 30 °C threshold recommended by the USP, which is the isothermal degree at which long-term stability was established for zone IVb conditions [23]. Though Qatar is categorized as being in zone IVa climate, the reference is still applicable as the temperature ranges for both zones (IVa and IVb) is the same. Assumptions can be made that 24-hour excursions within the acceptable limit would result in a negligible impact on drugs; however, this could be an oversight for medications which are thermolabile. The study findings align with previous literature. Studies from South Africa and Ohio reported MKT values in EMS environments that surpassed the recommended 25 °C of their region, emphasizing the need for EMS specific storage conditions [26, 27, 37].

The current study anticipated that the average daily MKT inside the RRCs would be greater compared to that of the bags (Fig. 5) due to the conductive nature of the metal net onto which the loggers were attached. This was true in RRC 1, 2, and 4, but not in RRC 3, 5, and 6 due to missing data in the scorching summer periods which resulted in potentially major temperature excursions not being recorded, decreasing the average in comparison to the measurements from the bags. Some data was also missing between April and July in bag 2 of RRC 6, which could justify why the readings inside this car were similar to those in the associated RRC bags. The error bars in this bar chart highlight the seasonal variability of the MKT averages.

Similarly, it was expected that the MKT violations inside the RRC exceeded those from the bags (Fig. 6). Like previously, missing readings in the summer in RRC 3, 5, and 6 resulted in fewer violations compared to other RRC’s, with the violations in RRC 6 being less than those of the associated bags (Fig. 7). Noticeably, the MKT violations ranged from 20 to 50% in most locations presenting a significant proportion of the data being outside the recommended ranges, highlighting the crucial need to assess the EMS drugs for their stability after being subjected to such harsh environmental conditions [24, 28].

Around six months of data was not reported from RRC 6 and so it is foreseen that the data does not meet expectations. Regarding RRC 3 and 5, even though they did have missing data, the values that they did have exceeded the recommended MKT by a significant degree to which they were able to raise the average more than that of the bags. All RRCs except RRC 4 had missing data, however those missing from RRC 1 and 2 were not significant to a degree that would affect the results like those of 3, 5, and 6. However, this is only an assumption until further inferential analysis is conducted.

This study offers several noteworthy strengths. First, it is the first of its kind in the Gulf region to continuously monitor real-life temperature and humidity conditions inside EMS rapid response vehicles over an entire year, capturing seasonal fluctuations and extreme summer conditions. Second, the use of duplicate data loggers in replicated medication bags allowed for validation of consistency and reliability across multiple data points. Third, calculating MKT on a daily basis provided a more accurate representation of cumulative thermal exposure, avoiding the masking effect of weekly or monthly averages used in previous studies.

However, some limitations due to logistical challenges faced in the EMS field should be considered. Due to operational constraints, the research medication bags were kept in the vehicle full-time, unlike actual paramedic bags which are routinely carried outside and hence exposed to more variable conditions. This may have underestimated the true environmental stress experienced during real shifts. In addition, paramedic bags were typically returned at the end of the shift to the ambulance station where they were restocked and temporarily stored in controlled conditions until they were endorsed to another crew; unlike in the study where the research medications bags were left in the RRC the whole time in uncontrolled conditions. Additionally, missing data due to logger memory limits or manual stoppage, particularly in some RRCs during peak summer, may have introduced bias and reduced completeness. Finally, the study did not assess the chemical stability or pharmacological efficacy of the drugs exposed to these excursions, which limits the ability to directly link environmental exposure to clinical impact. Although possible drug degradation under these conditions is yet to be assessed, it is important to develop and implement strategies, which allow for more stringent storage environments to be maintained. Such implementations could include the utilization of real-time monitoring systems to alert paramedics of any deviations within drug storage compartments. Other strategies may include educating paramedics on appropriate drug storage conditions or investing in appropriate medication storage compartments within the ambulance.

Improved data collection to minimize missing data, bias, errors, and improve generalizability can be done by using telemetric sensors or more than one data logger in each setting, or by using advanced temperature measuring devices such as time-temperature indicators (TTIs) which may be advantageous in monitoring temperature, as well as being smaller, more affordable, and simpler in use when compared to other monitors [29]. Ensuring that paramedics on shift manage the loggers at the start of the shift or even weekly may be useful. Future studies could expand the focus to other environmental parameters (ex: light and agitation) and the study design can be adapted to different climate zones, allowing for region-specific storage guidelines based on temperature and humidity. Studies comparing the efficacy of different available storage conditions or equipment would also prove vital in this field. While our current analysis does not stratify data based on time of day and specific vehicle routes, future studies could explore temporal trends and route-specific effects to better understand any nuanced patterns in the data. In general, this methodology and presentation approach of the results can be utilized in similar future research conducted in varying climates.

## Conclusion

Findings showed that during summer, temperatures in rapid response cars exceeded those recommended by the guidelines, subjecting medications to stress conditions, potentially risking their integrity. The MKT results indicated alarming excursions, which should only be used as an indicator of the severity of the current storage conditions and not to normalize them, highlighting the need to reassess the current medication storage conditions in ambulances within climate zone IV regions and their effect on drug safety and efficacy.

## Electronic supplementary material

Below is the link to the electronic supplementary material.

Supplementary Material 1

## Data Availability

All data generated or analysed during this study are included in this published article [and its supplementary appendix file].

## Abbreviations

EMS: Emergency Medical Services
MKT: Mean Kinetic Temperature
HMCAS: Hamad Medical Corporation Ambulance Service
RH: Relative Humidity
ICH: International Council for Harmonisation of Technical Requirements for Pharmaceuticals for Human Use
WHO: World Health Organization
EMA: European Medicine Agency
USP: United States Pharmacopeia
MENA: Middle East and North Africa
RRC: Rapid-Response Car
USL: Upper Specification Limit
CV: Coefficient of Variance
TTIs: Time-Temperature Indicators
