# Supplementary material for: Time-based tracking of temperature and humidity of emergency medical service rapid response vehicles in Qatar: a prospective observational study
Source: BMC Emerg Med. 2025 Jul 1;25:98. doi: 10.1186/s12873-025-01255-3 (PMC12211764; doi:10.1186/s12873-025-01255-3)
Supplement: Supplementary file 1 — Supplementary Material 1 [file 12873_2025_1255_MOESM1_ESM.docx]

**Appendix 1: Medications commonly stored in the paramedic bags of the rapid response cars in Hamad Medical Corporation Ambulance Service (HMCAS) and their recommended storage conditions and usage data.**

| **No** | **Medication** | **Dosage Form*** | **Manufacturer Storage Recommendations** | **HMCAS Average Monthly Usage in Numbers (min-max)** |
| --- | --- | --- | --- | --- |
| 1 | Adenosine 6mg/2ml | UDV | Store below 25°C in a cool, dry place. Do not refrigerate. | 63 (38-75) |
| 2 | Adrenaline 1mg/10ml | PFS | Do not store above 25°C. | 375 (225-450) |
| 3 | Amiodarone 150mg/3ml | Amp | Do not store above 25°C. Do not refrigerate or freeze. | 50 (30-60) |
| 4 | Atropine 0.5mg/1ml | PFS | Store at 20-25°C. Excursions permitted between 15°C and 30°C. | 50 (30-60) |
| 5 | Calcium chloride 10%/10ml | PFS | Store at 20-25°C. | 25 (15-30) |
| 6 | Diclofenac 75mg/3ml | Amp | Store below 30°C. | 63 (38-75) |
| 7 | Diphenhydramine 50mg/1ml | Vial | Store at 20-25°C. | 63 (38-75) |
| 8 | Fentanyl 100mcg/2ml | Amp | Store below 25°C. | 250 (188-375) |
| 9 | Furosemide 20mg/2ml | Amp | Do not store above 25°C. | 31 (19-38) |
| 10 | Glyceryl trinitrate 50mg/10ml | Amp | Store below 25°C. Protect from light. | 63 (38-75) |
| 11 | Hydrocortisone 100mg | Pwd | Store unreconstituted product at controlled room temperature 20 -25°C | 188 (113-225) |
| 12 | Ibuprofen 400mg | Tab | Store below 30°C. | 3750 (2250-4500) |
| 13 | Ipratropium 250mcg/2ml | UDV | Unopened bottles should be stored at controlled room temperature at 15-30ºC. Protect from light. | 250 (150-300) |
| 14 | Ketamine 500mg/10ml | UDV | Store at 15-30ºC and at ambient relative humidity. Protect from light. | 150 (113-225) |
| 15 | Magnesium sulphate 2g/20ml | Vial | Store at 20-25°C. Do not freeze. | 88 (53-105) |
| 16 | Metoclopramide 10mg/2ml | PFS | Store at 20° to 25°C. Protect from light. | 25 (15-30) |
| 17 | Midazolam 15mg/3ml | Amp | Store below 30°C. | 50 (38-75) |
| 18 | Naloxone 400mcg/1ml | Amp | Store below 25°C. Dilutions should be used immediately. If not used immediately, in-use storage times and conditions prior to use are the responsibility of the user and would normally not be longer than 24 hours at 2-8°C, unless dilution has taken place in controlled and validated aseptic conditions. | 38 (23-45) |
| 19 | Ondansetron 4mg/2ml | Amp | Store below 30°C (Store at 20-25°C for vials). Do not freeze. Protect from light. It must be stored at 2-8°C for not more than 24 hours. It does not need to be protected from light during infusion. | 63 (38-75) |
| 20 | Oral glucose 31g | Gel | Store in a closed container at room temperature, away from heat, moisture, and direct light. Do not freeze. | 125 (75-150) |
| 21 | Paracetamol 1g/100ml | UDV | Do not store above 25ºC. Do not refrigerate or freeze. | 1500 (900-1800) |
| 22 | Paracetamol 500mg | Tab | Store below 25°C. Protect from moisture. | 5000 (3000-6000) |
| 23 | Phenylephrine 10mg/1ml | Amp | Do not store above 30ºC Protect from light. | 63 (38-75) |
| 24 | Rocuronium 50mg/5ml | UDV | Store at 2-8°C. The product can be stored outside the refrigerator at a temperature of up to 30°C for a maximum of 3 months. | 188 (113-225) |
| 25 | Salbutamol 0.5mg/1ml | Amp | Store at 15-25ºC. Protect from light. | 4 (2-5) |
| 26 | Salbutamol 2.5mg/3ml | UDN | Store below 30°C. Protect from light. | 625 (375-750) |
| 27 | Sodium chloride 0.9%/10ml | Amp | Store below 25°C. | 63 (38-75) |
| 28 | Suxamethonium 100mg/2ml | Amp | Store at 2°C to 8°C. Do not freeze. | 13 (8-15) |
| 29 | Tranexamic acid 0.5g/5ml | Amp | Store below 25°C. Do not freeze. Protect from light. | 63 (38-75) |

**Amp: ampule, Pwd: powder, PFS: prefilled syringe, Tab: tablet, UDN: unit dose nebule, UDV: unit dose vial.*

**Appendix 2: Percentage of the 10-min intervals over 12 months during which temperature or relative humidity readings exceeded the guideline recommendations of 30°C and 65%, respectively, for each rapid response car.**

| **Logger location**  **(Vehicle and bag number)** | **Percentage of time intervals when the temperature exceeded the maximum recommendation of 30 °C** | **Percentage of time intervals when the relative humidity exceeded the maximum recommendation of 65%** |
| --- | --- | --- |
| Rapid Response Car 1, bag 1 | 23.7 % | 1.4 % |
| Rapid Response Car 1, bag 2 | 23.4 % | 1.4 % |
| Rapid Response Car 1 | 34.9 % | 1.2 % |
| Rapid Response Car 2, bag 1 | 33.7 % | 0.8 % |
| Rapid Response Car 2, bag 2 | 29.3 % | 0.7 % |
| Rapid Response Car 2 | 39.1 % | 1.5 % |
| Rapid Response Car 3, bag 1 | 27.2 % | 1.0 % |
| Rapid Response Car 3, bag 2 | 29.7 % | 1.0 % |
| Rapid Response Car 3 | 15.2 % | 1.4 % |
| Rapid Response Car 4, bag 1 | 30.7 % | 0.8 % |
| Rapid Response Car 4, bag 2 | 29.7 % | 0.9 % |
| Rapid Response Car 4 | 32.3 % | 1.7 % |
| Rapid Response Car 5, bag 1 | 22.3 % | 0.3 % |
| Rapid Response Car 5, bag 2 | 20.4 % | 0.5 % |
| Rapid Response Car 5 | 21.5 % | 1.6 % |
| Rapid Response Car 6, bag 1 | 30.9 % | 1.1 % |
| Rapid Response Car 6, bag 2 | 27.3 % | 1.4 % |
| Rapid Response Car 6 | 26.0 % | 1.1 % |

**Appendix 3: Temperature readings for each 10-min interval over 12 months from the different drug storage locations. Each source is presented separately in a graph (a-r). Dotted red line is the upper specification limit (USL) of 30 °C. Possible reasons leading to missing data have been identified as indicated in the inserted boxes.**

**
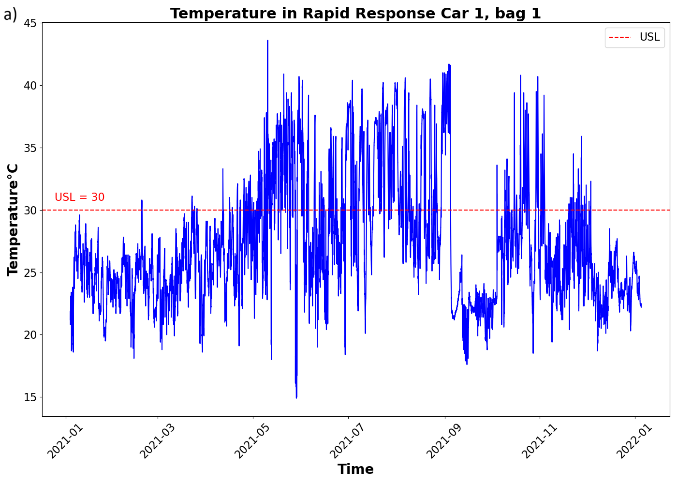

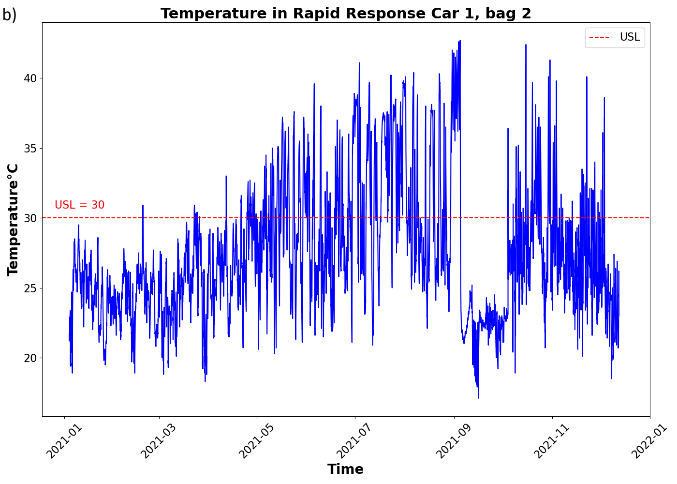

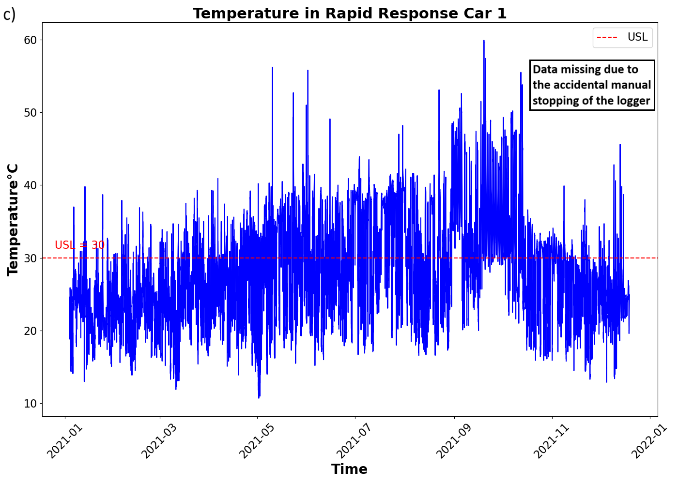

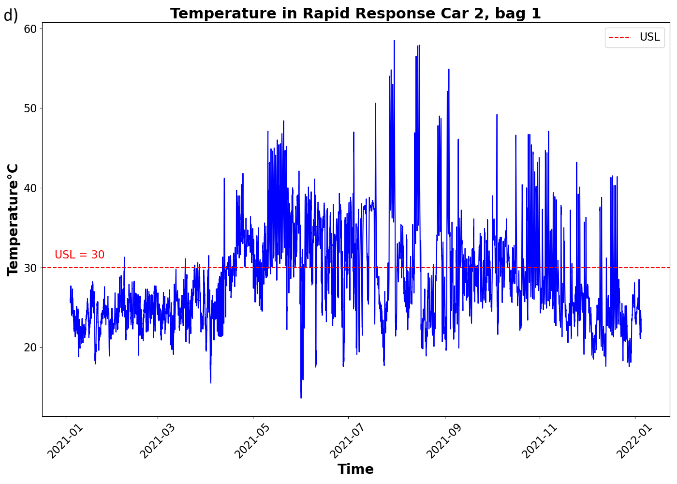

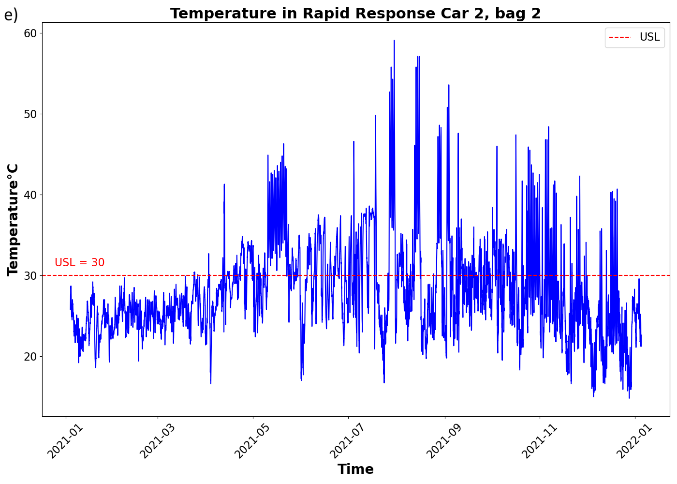

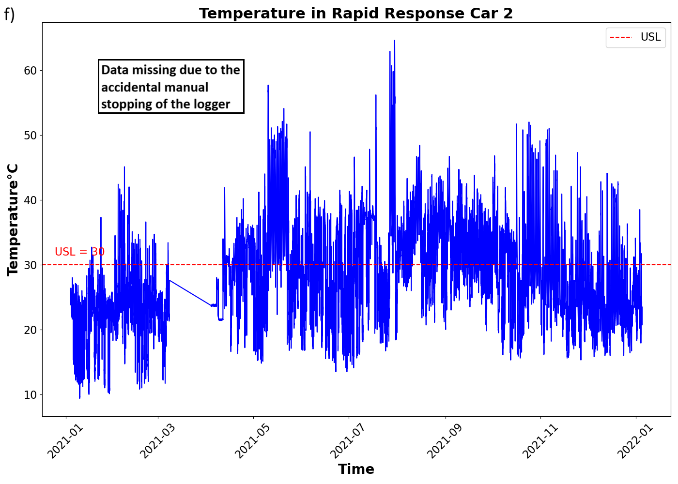

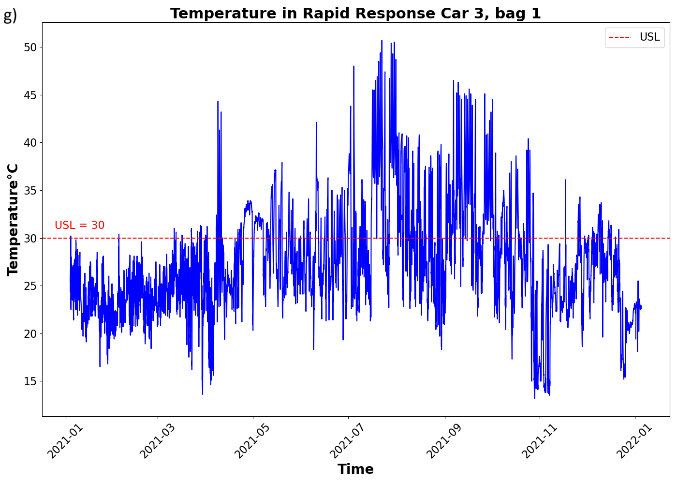

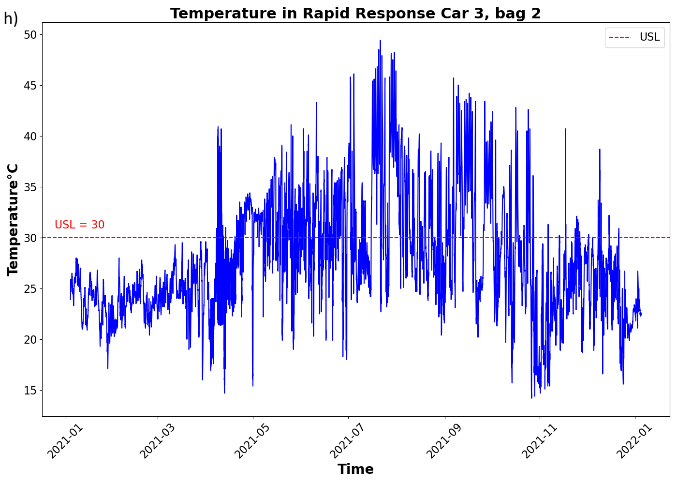

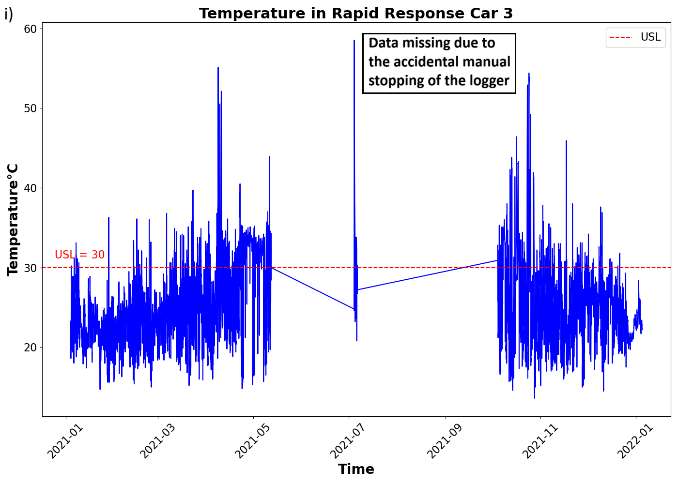

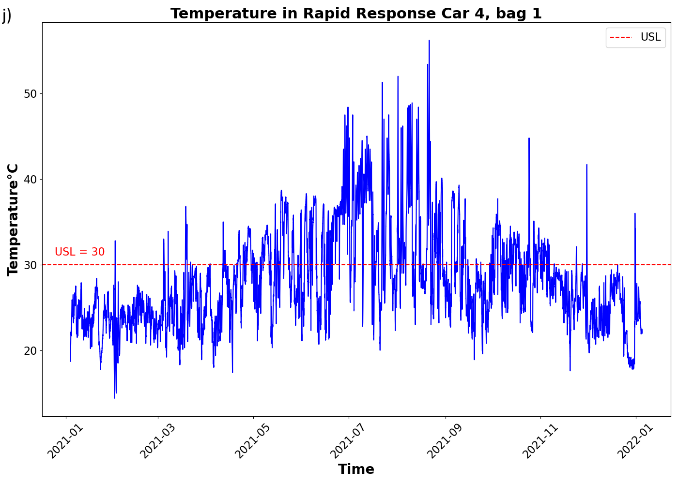

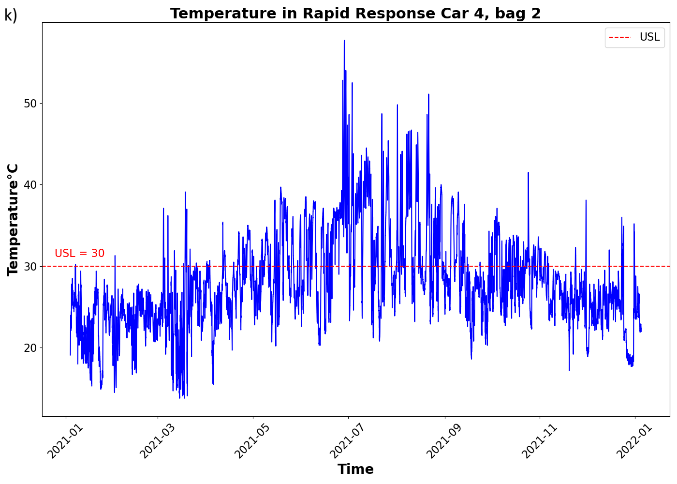

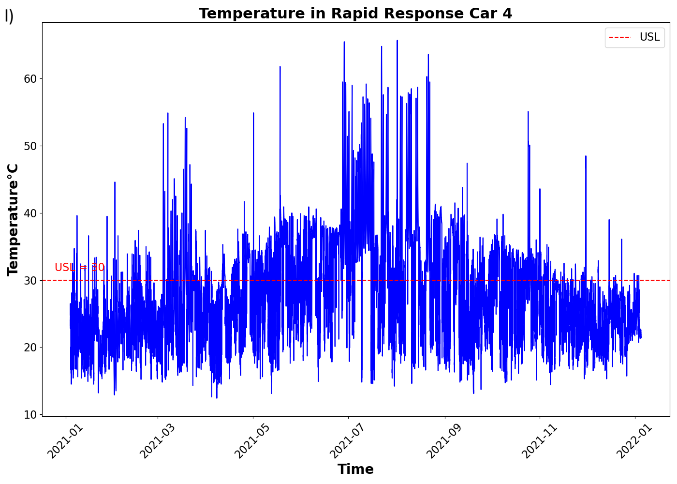

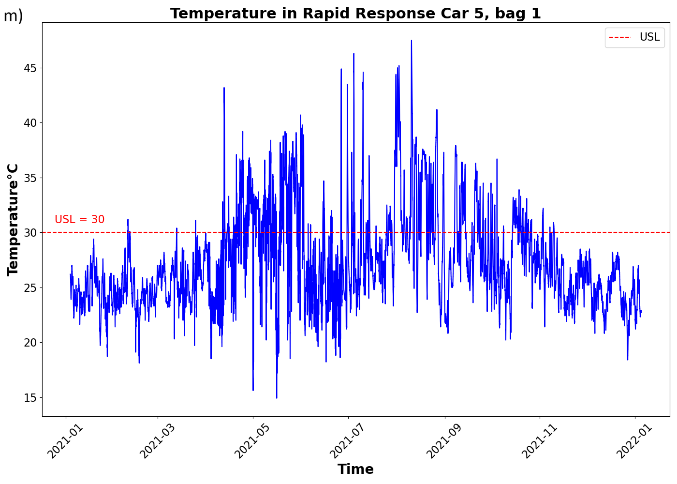

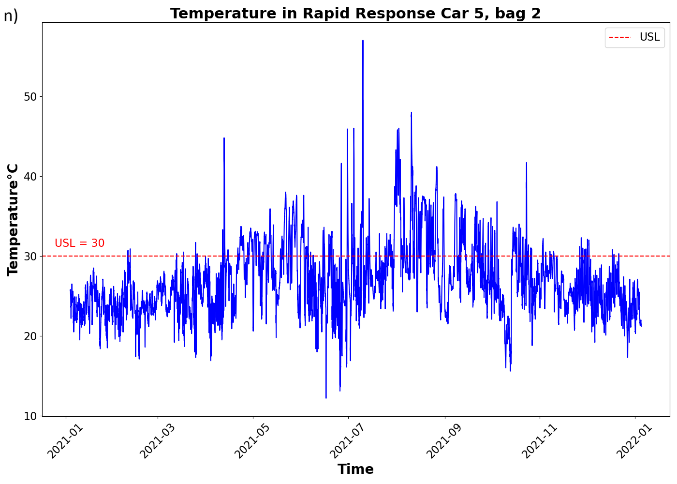

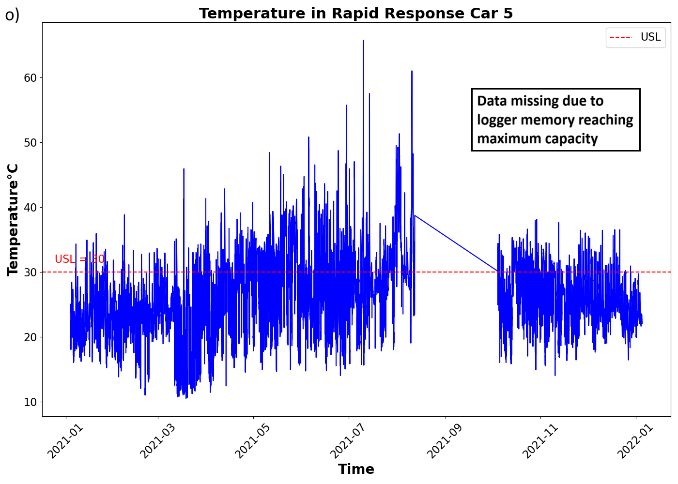

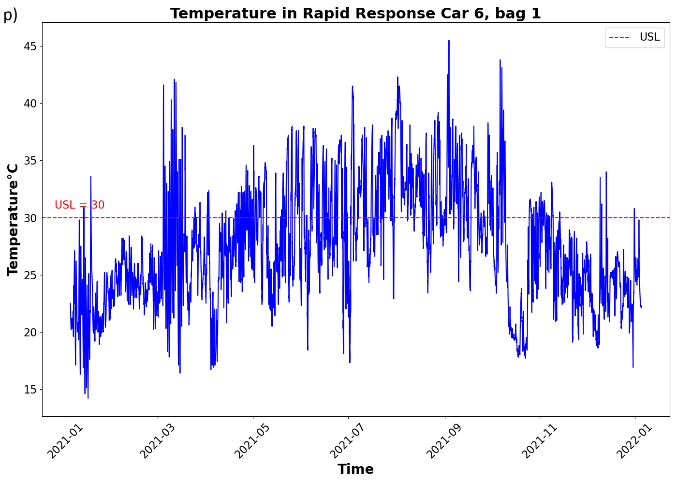

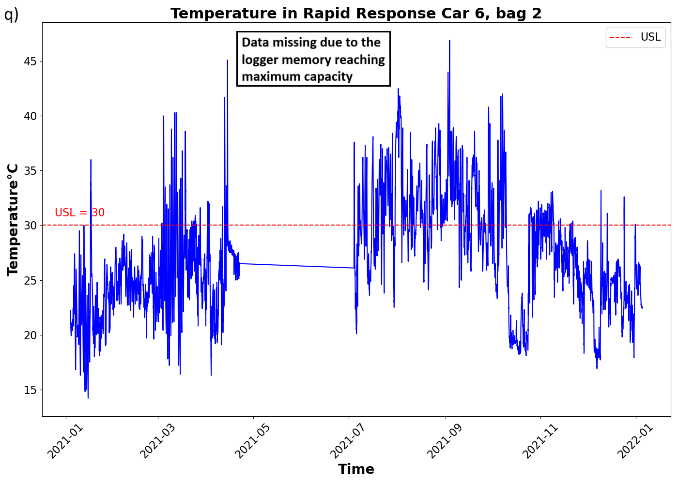

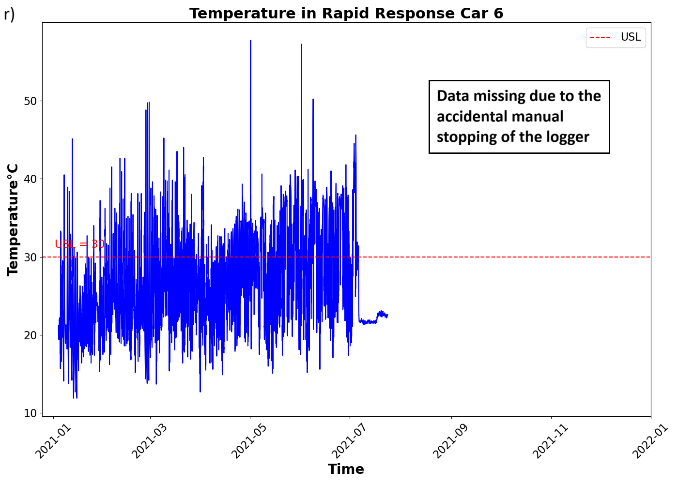
**

**Appendix 4: Relative humidity readings for each 10-min interval over 12 months from the different drug storage locations. Each source is presented separately in a graph (a-r). Dotted red line is the upper specification limit (USL) of 65% RH. Possible reasons leading to missing data have been identified as indicated in the inserted boxes.**

**
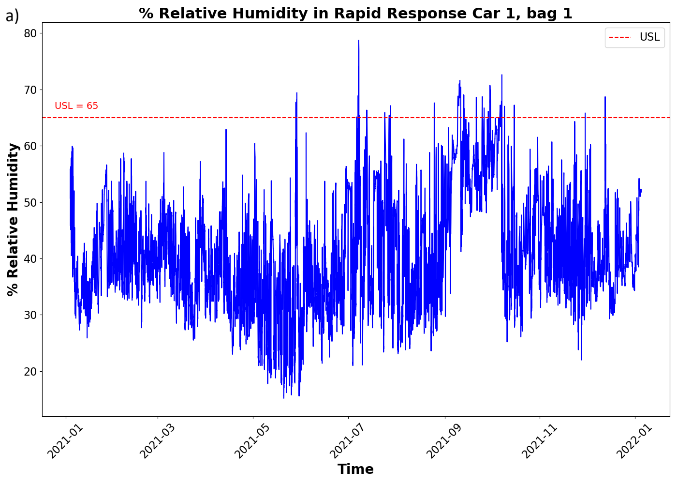

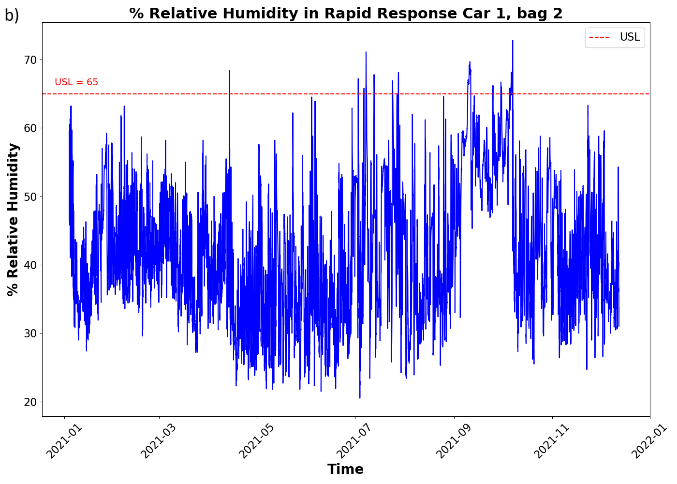

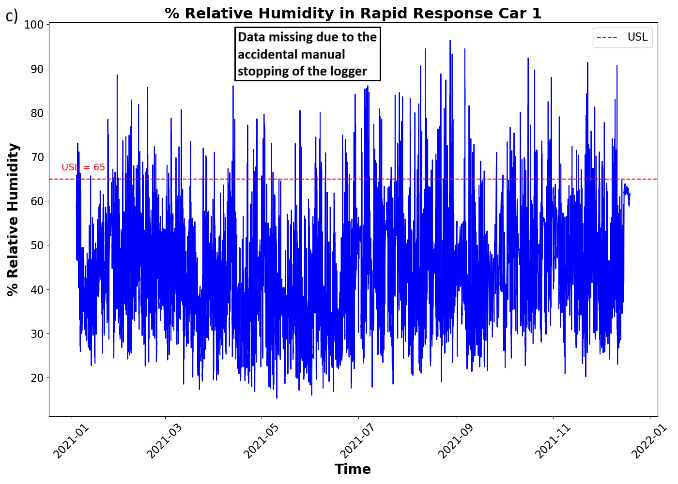

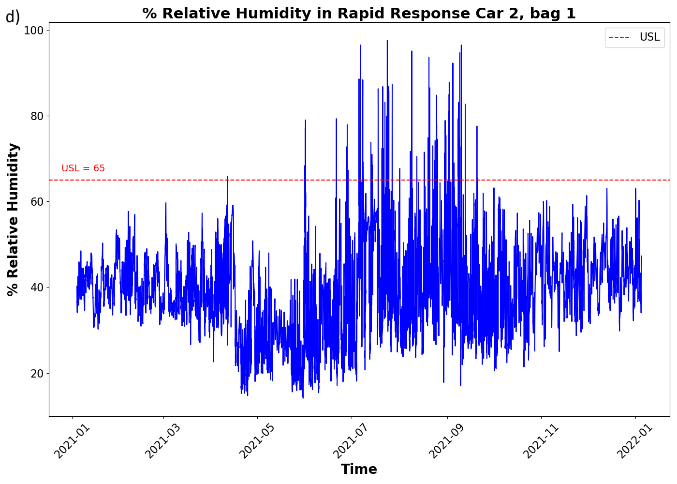

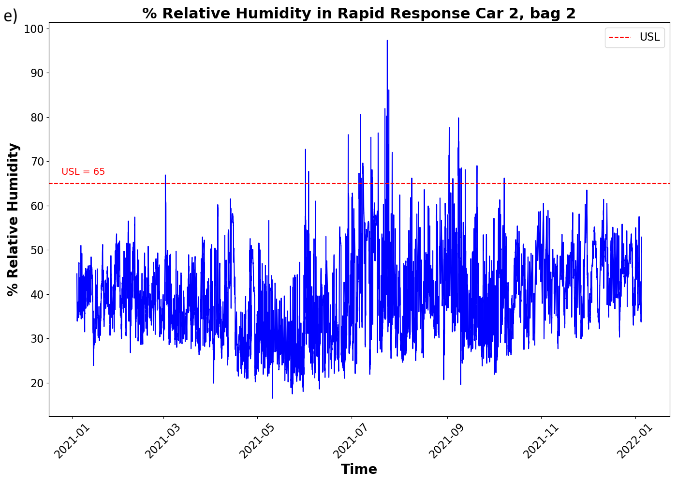

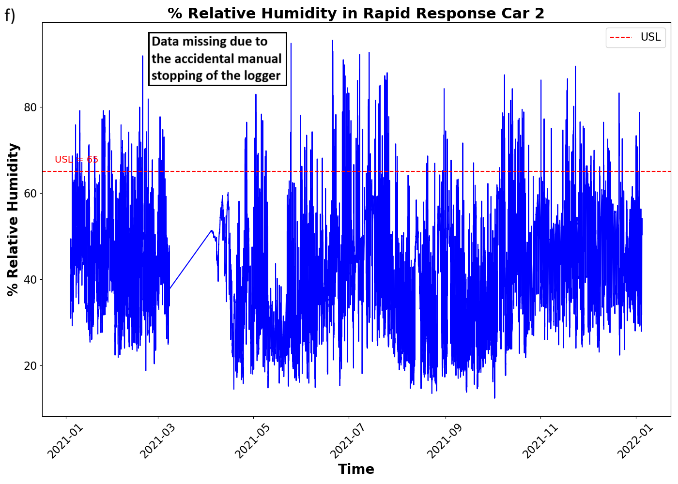

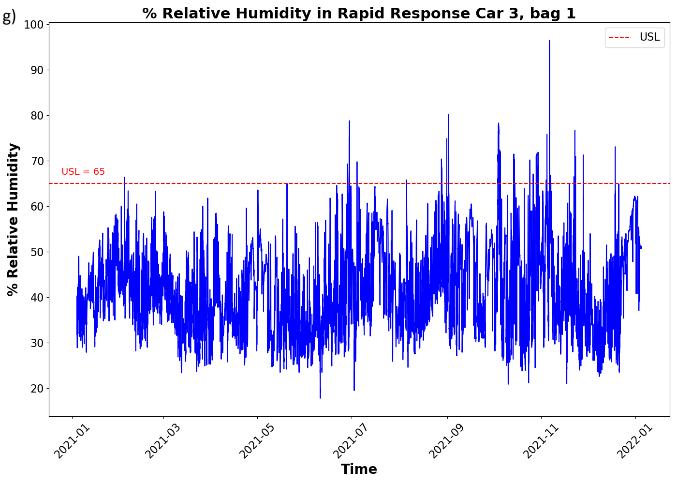

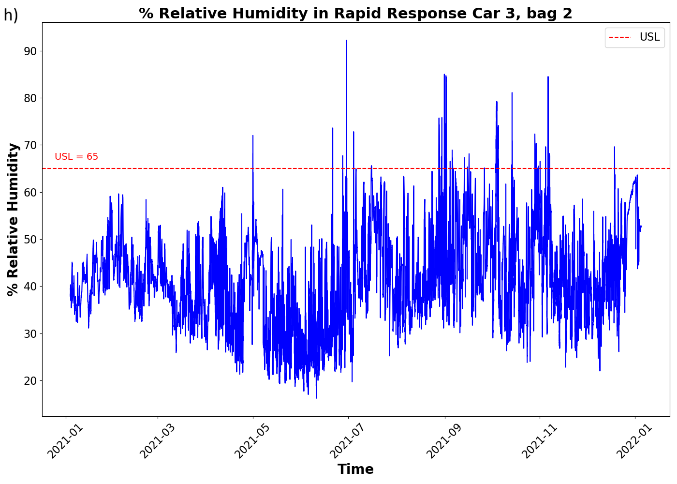

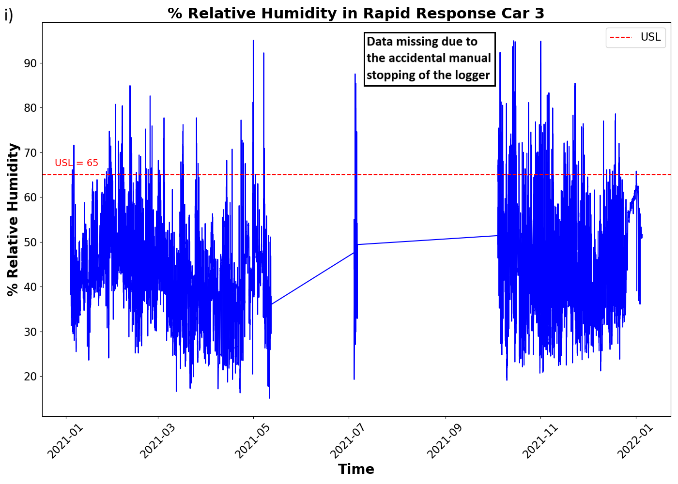

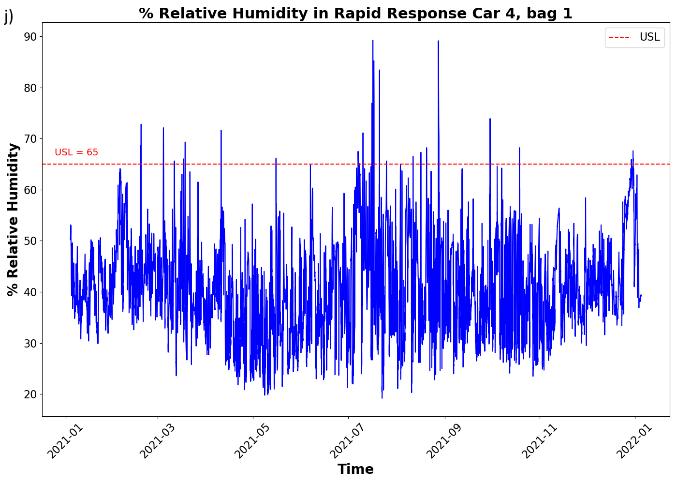

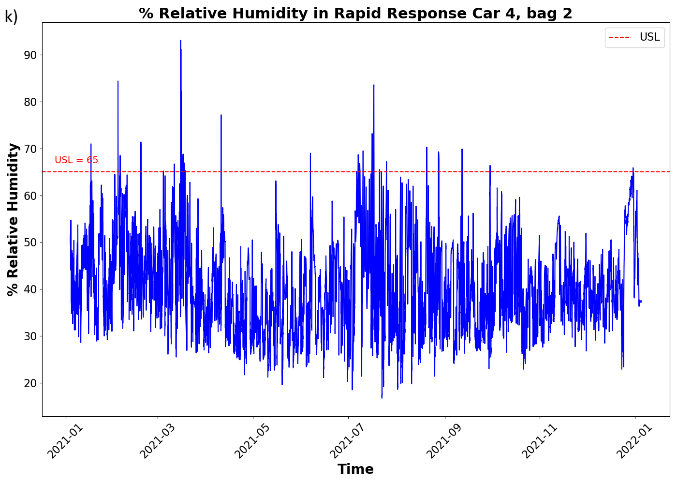

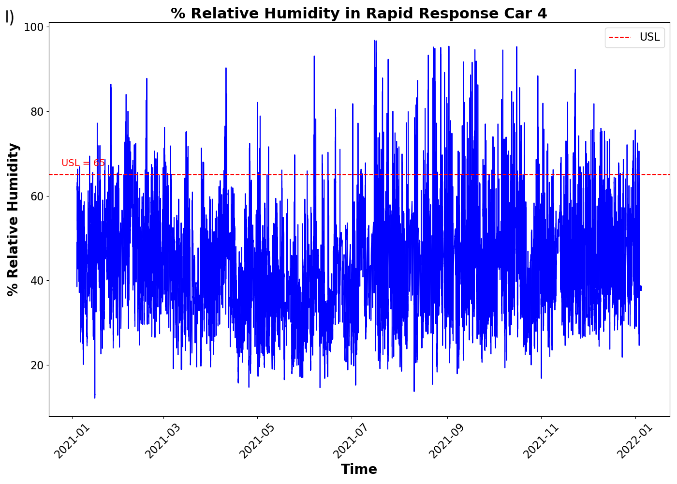

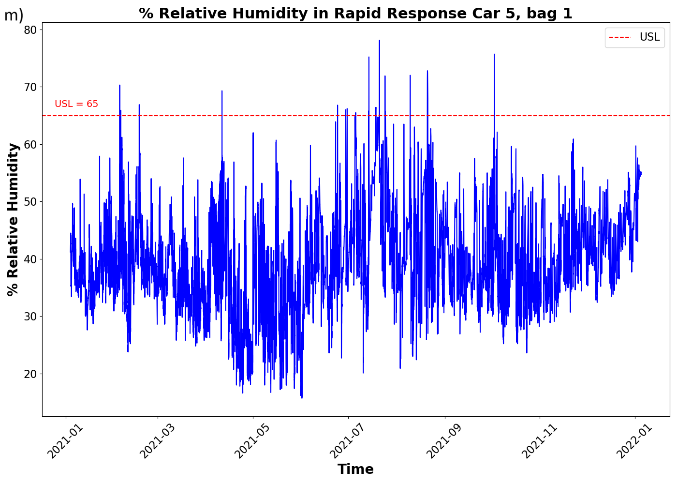

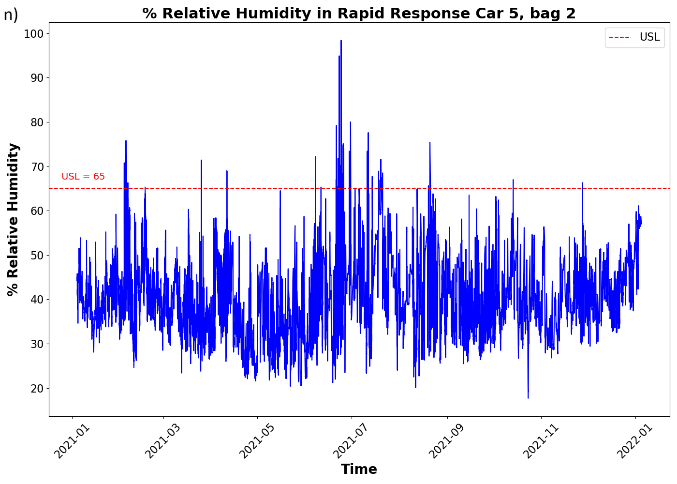

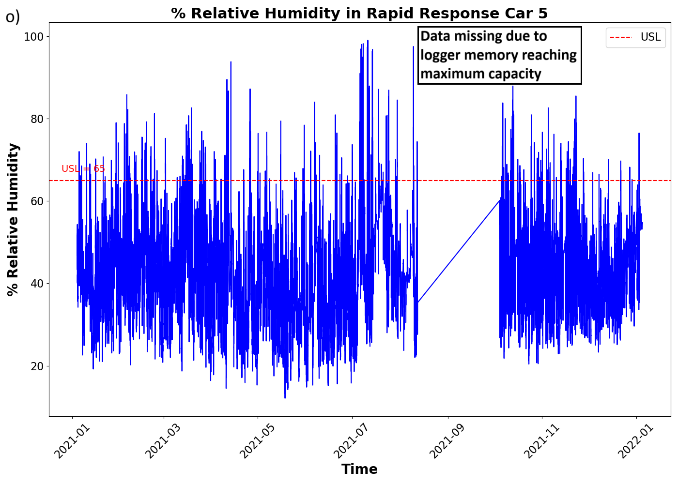

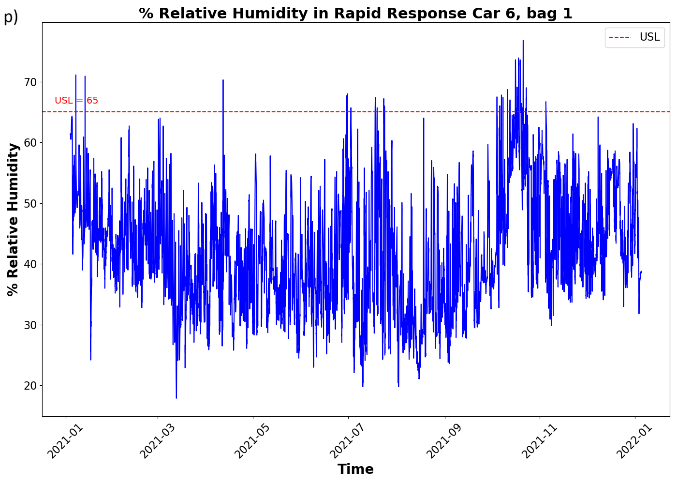

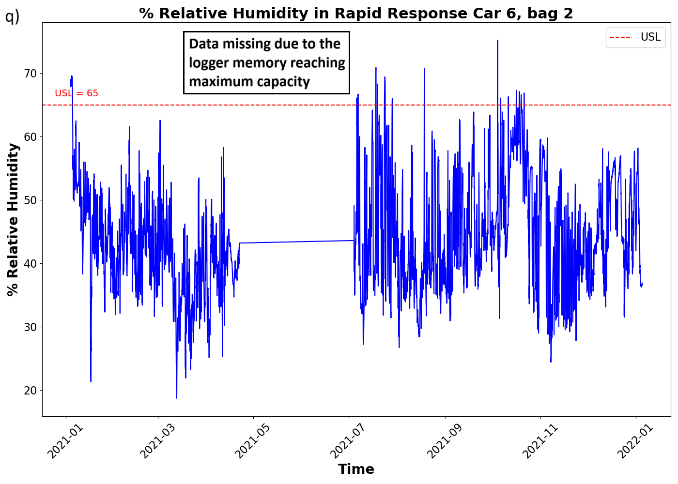

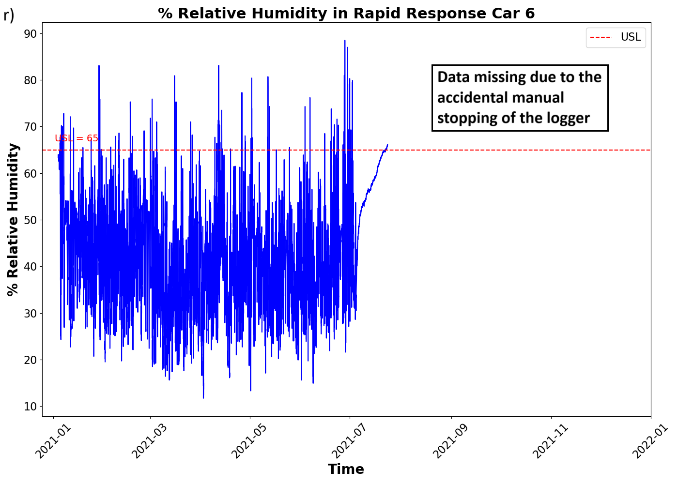
**

**Appendix 5: Daily mean kinetic temperature values reported from each drug storage location. Each source is presented separately in a graph (a-r). Dotted red line is the upper specification limit (USL) of 30 °C. Possible reasons leading to missing data have been identified as indicated in the inserted boxes.**

**
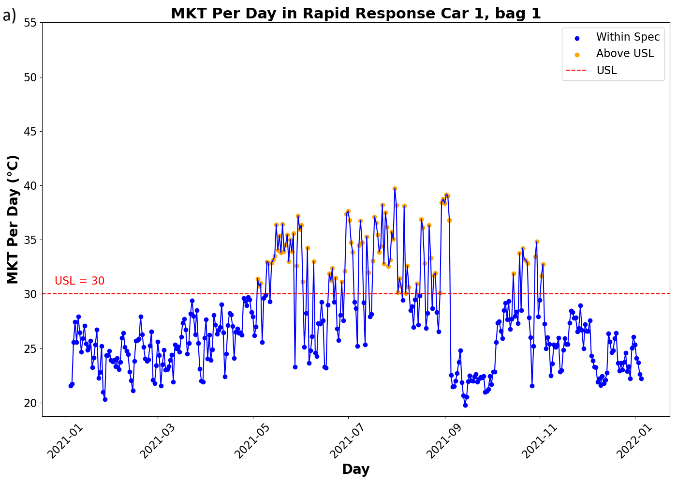

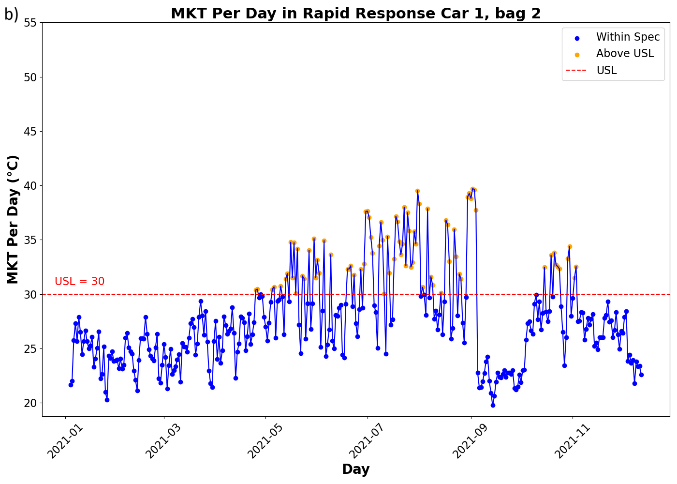

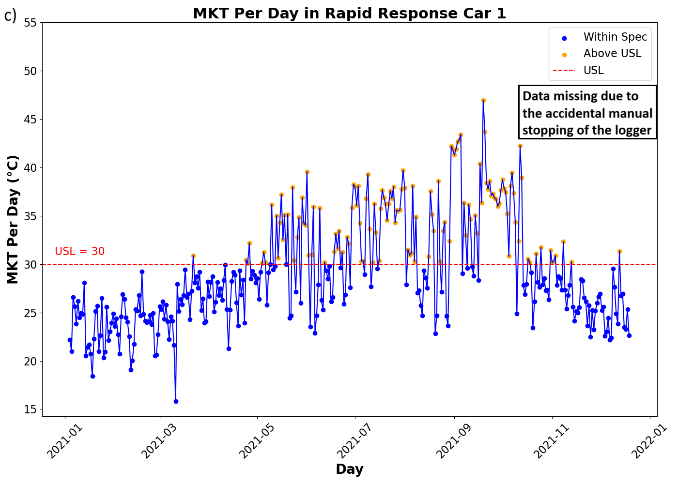

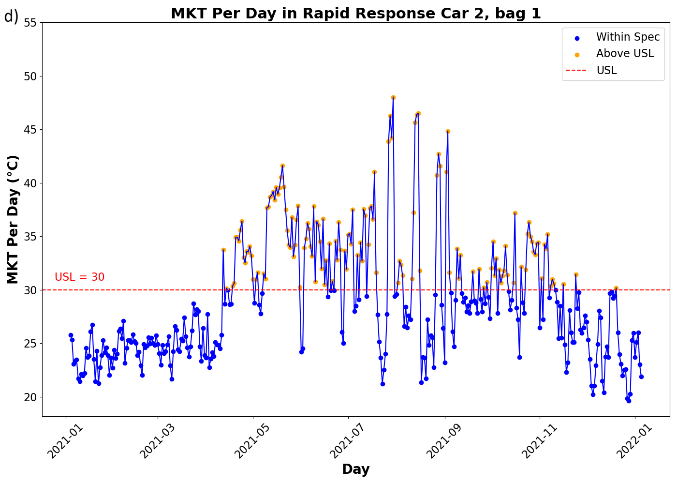

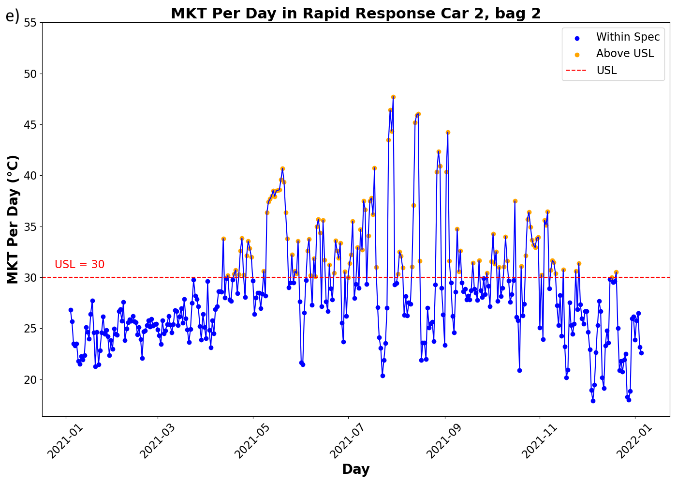

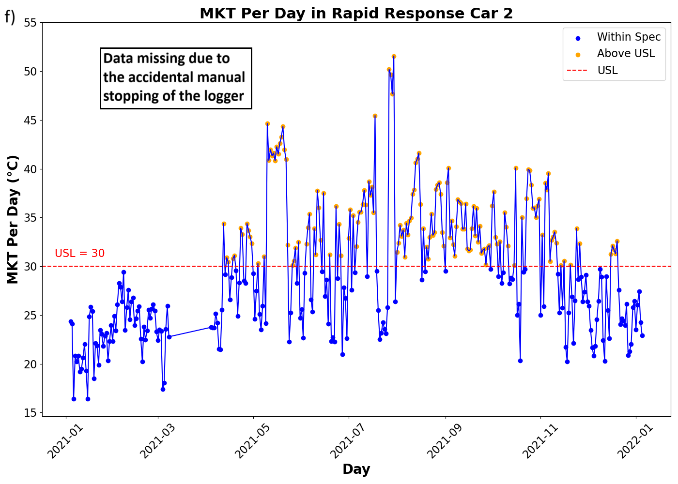

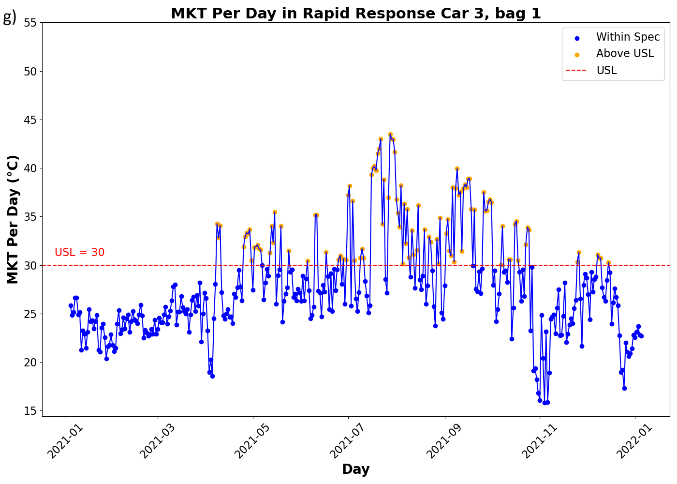

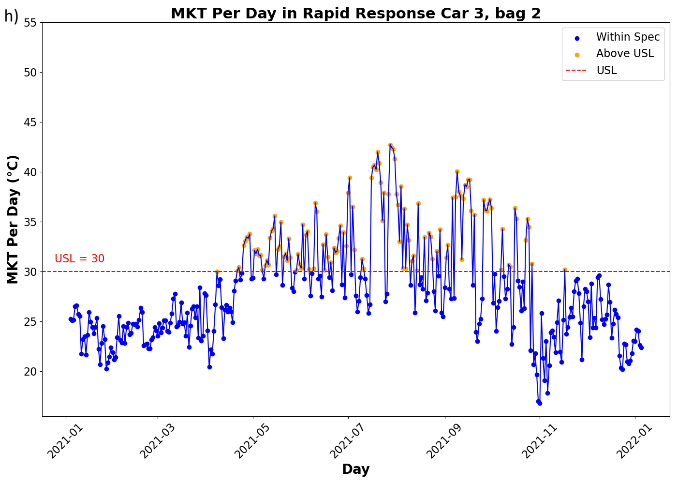

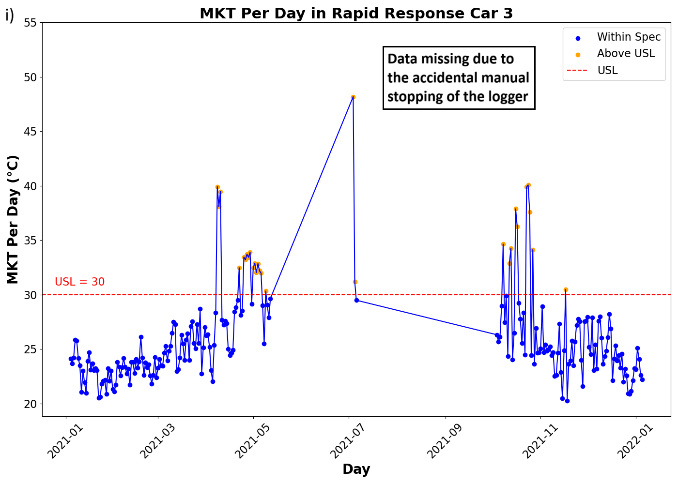

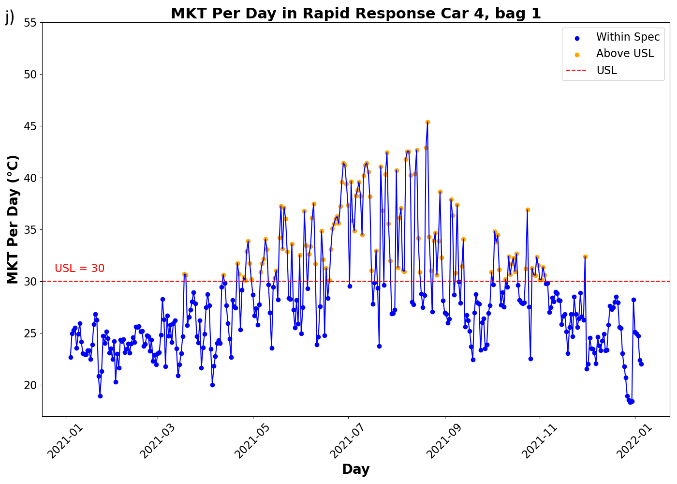

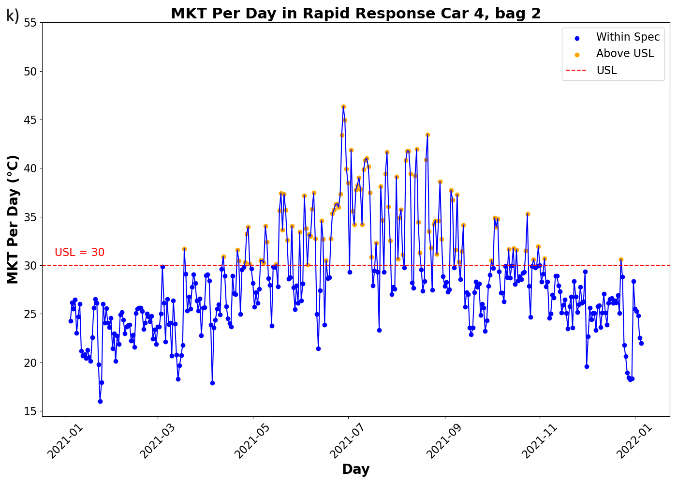

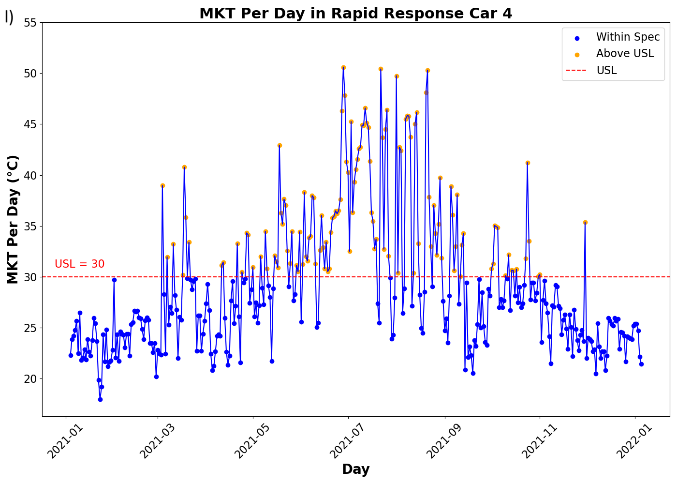

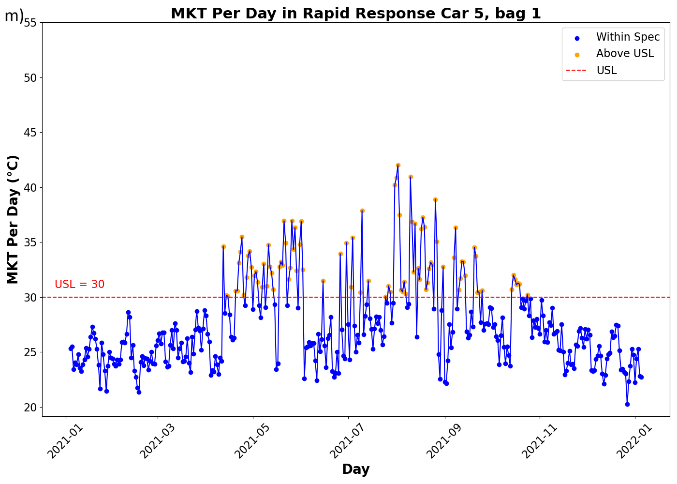

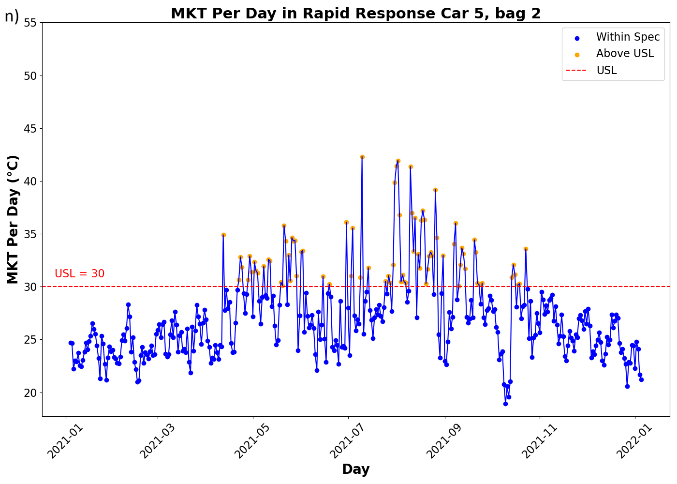

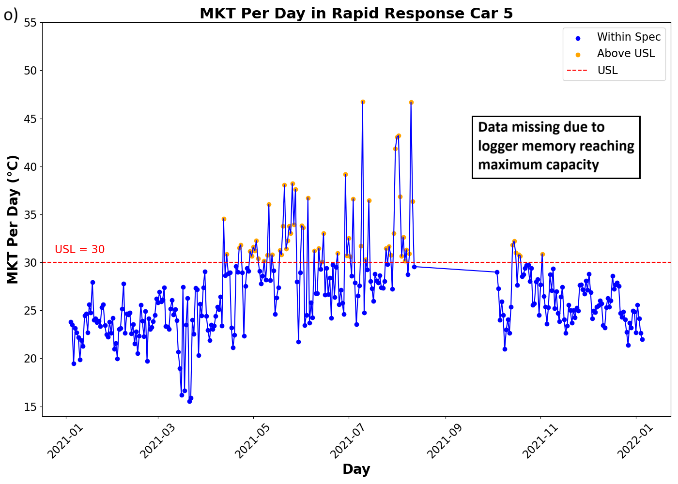

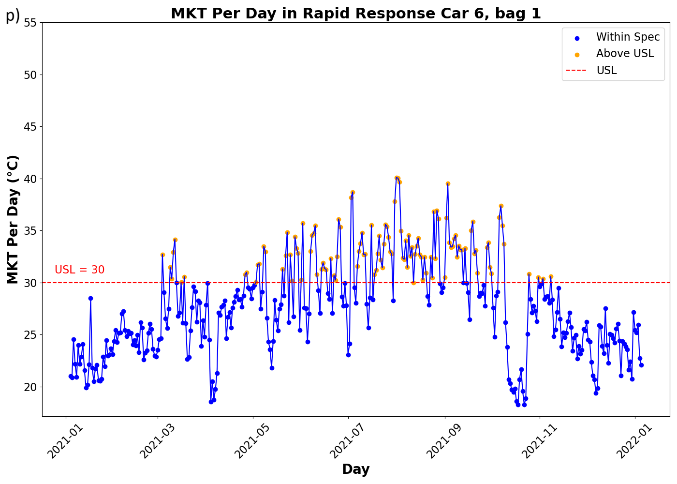

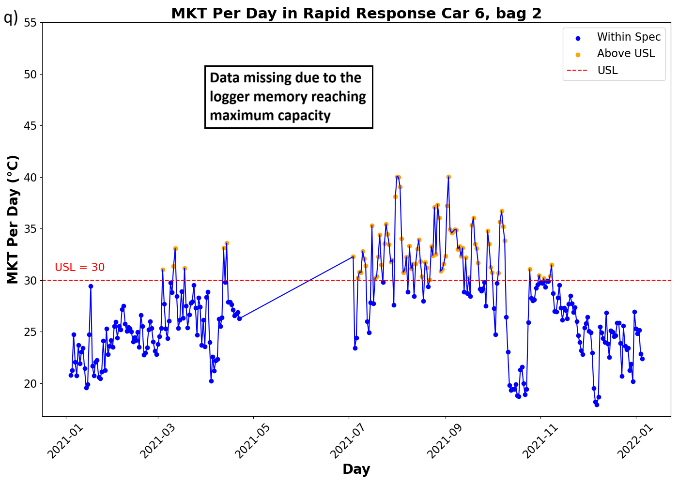

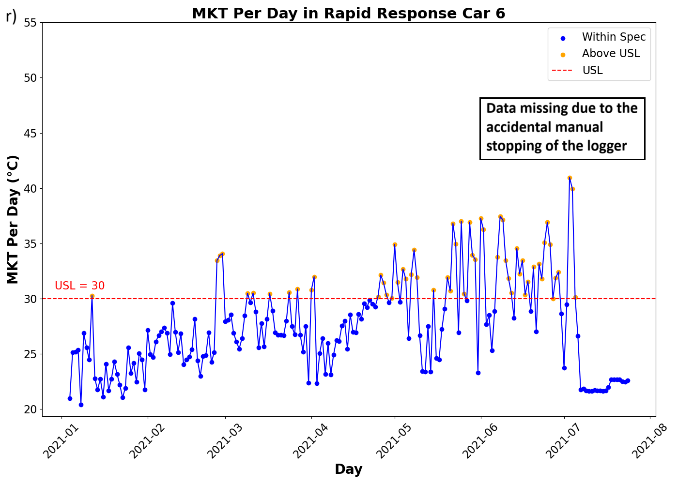
**

**Appendix 6: Average of daily mean kinetic temperature values reported over a month from all drug storage locations for each vehicle. Each month is presented separately in a graph (a-m). Dotted red line is the upper specification limit (USL) of 30 °C.**

**
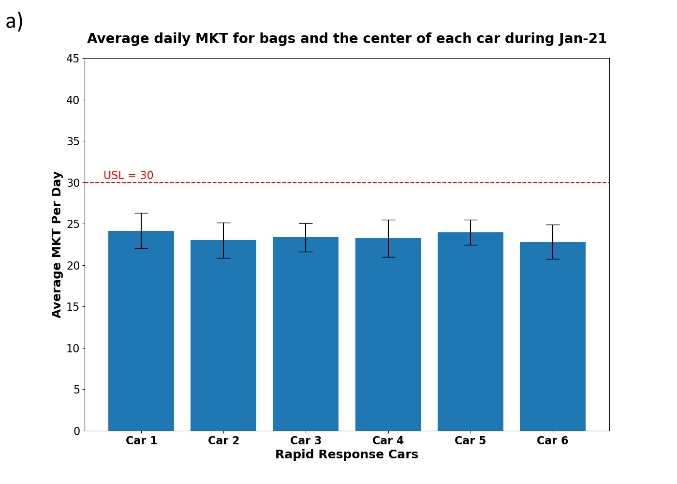

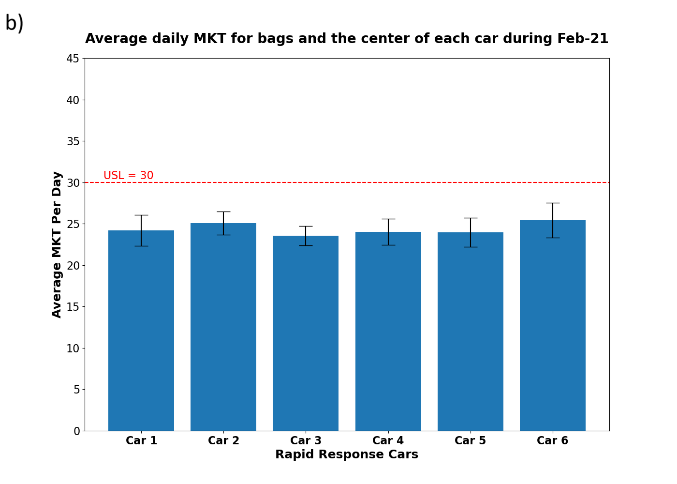

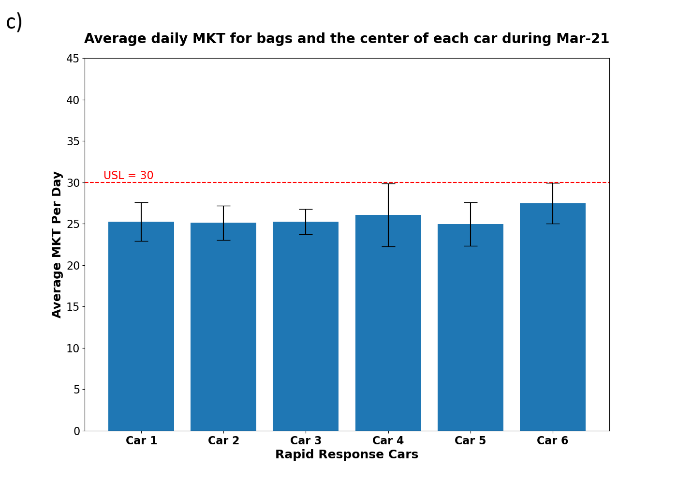

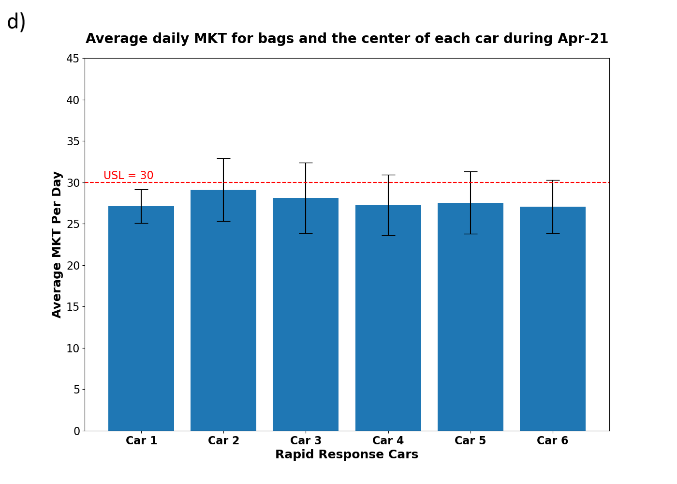

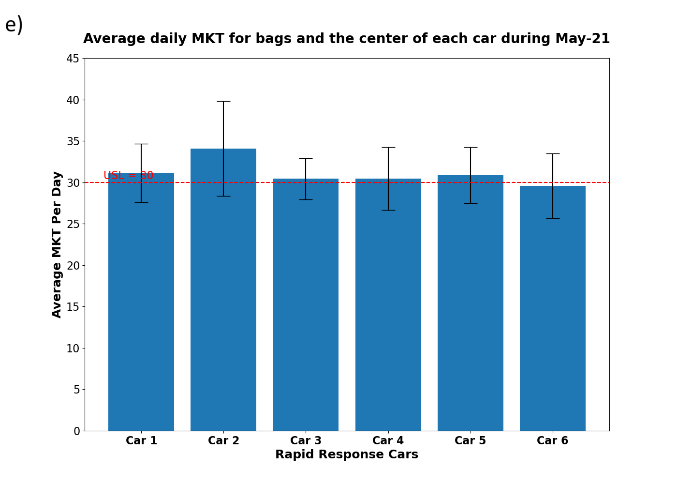

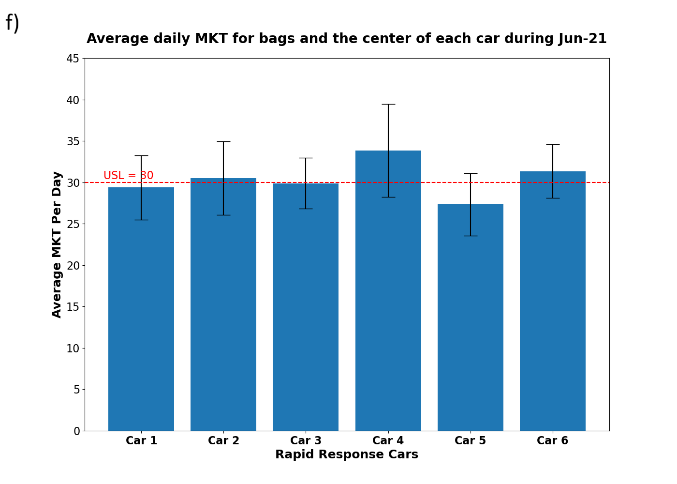

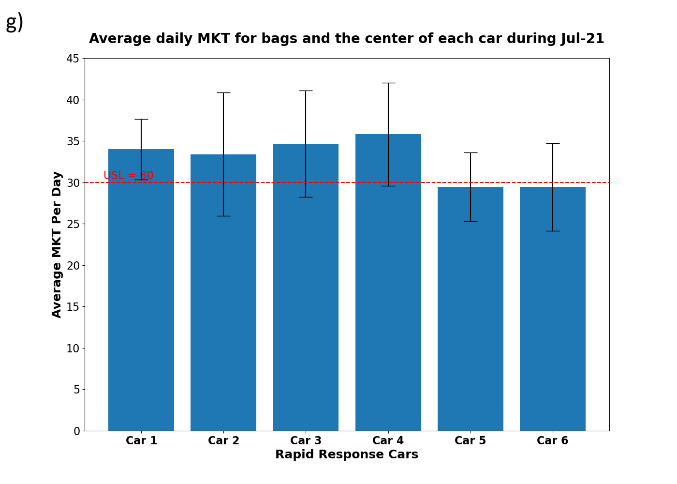

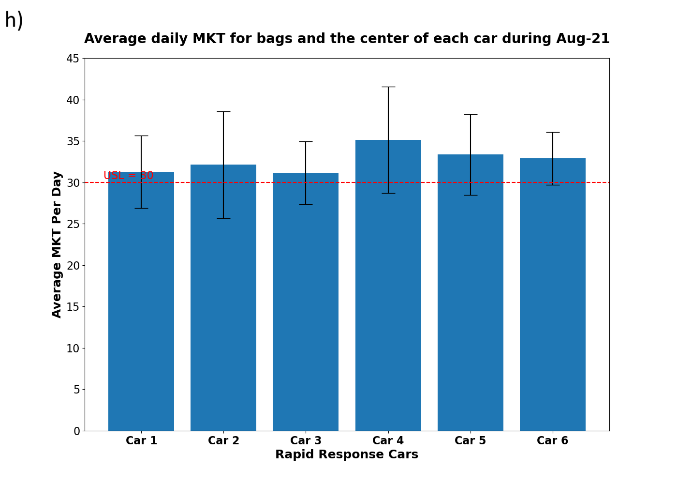

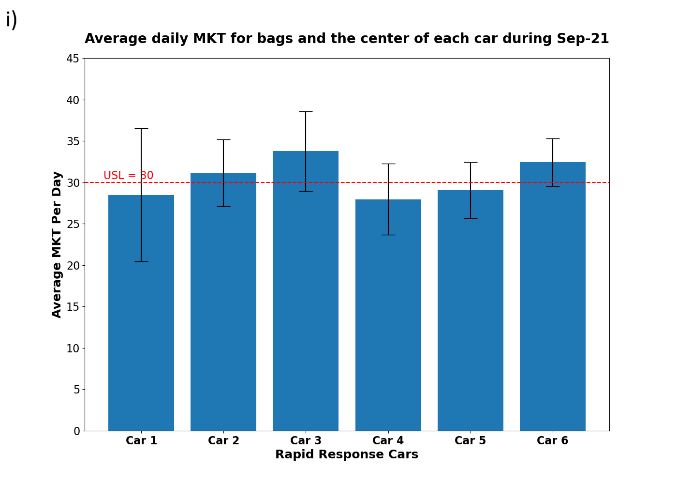

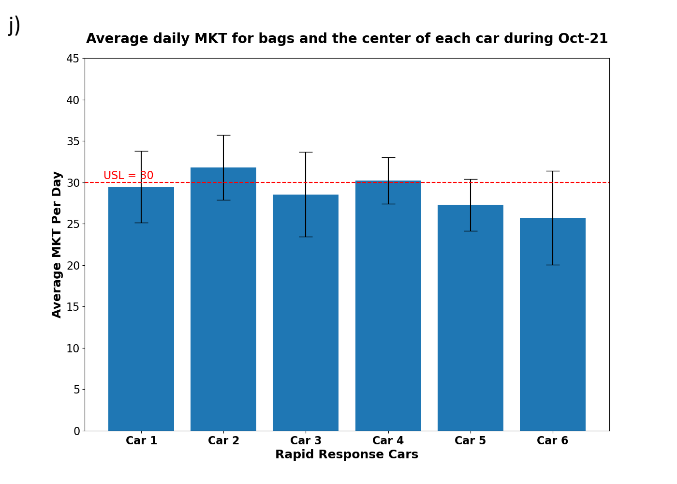

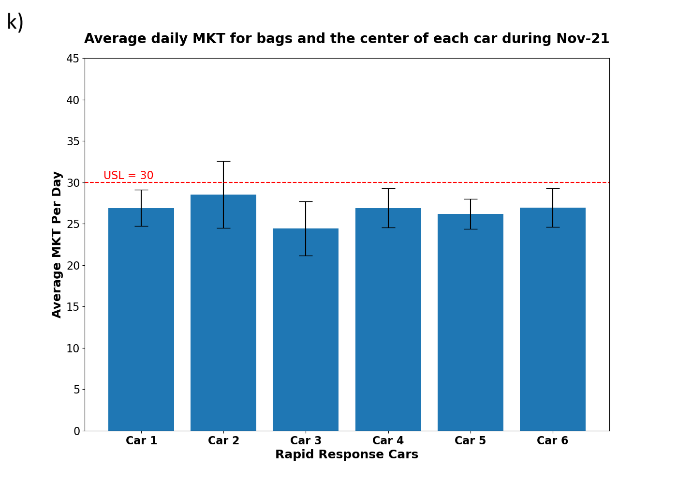

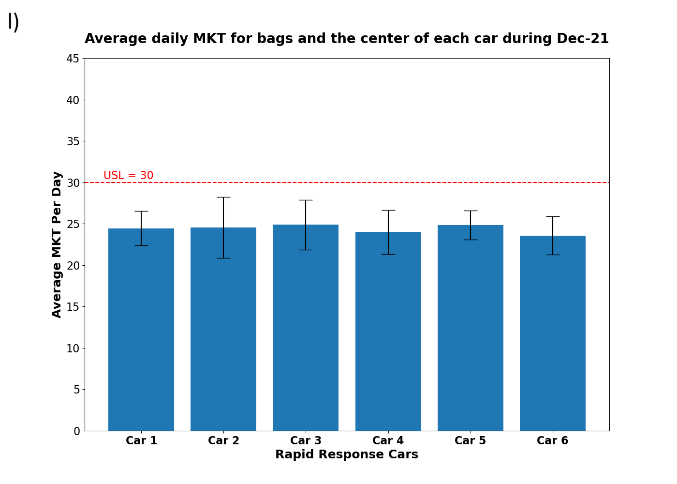
**
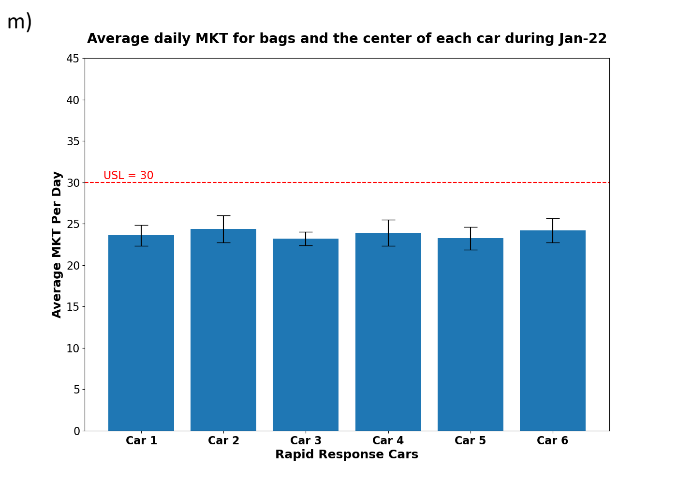


**Appendix 7: Average of daily mean kinetic temperature values reported over a month from bags only for each vehicle. Each month is presented separately in a graph (a-m). Dotted red line is the upper specification limit (USL) of 30 °C.**

**
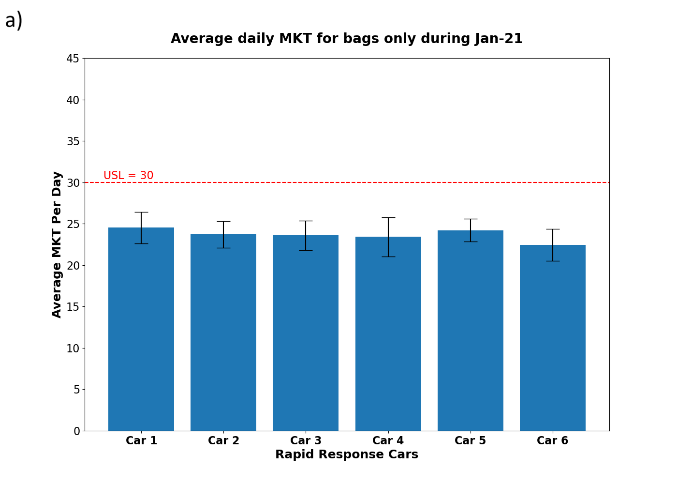

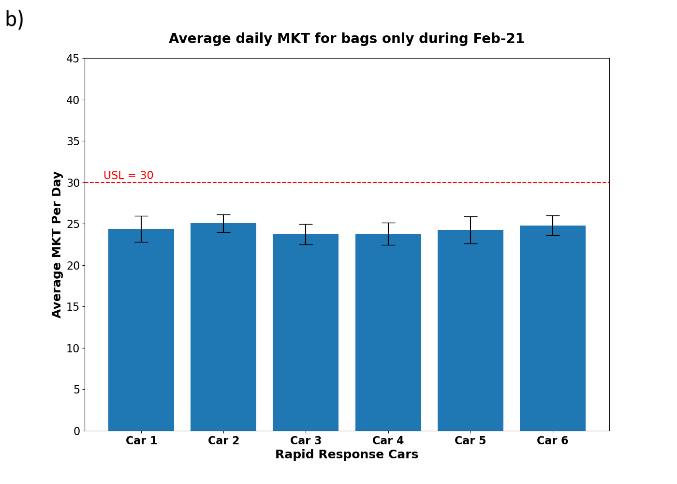

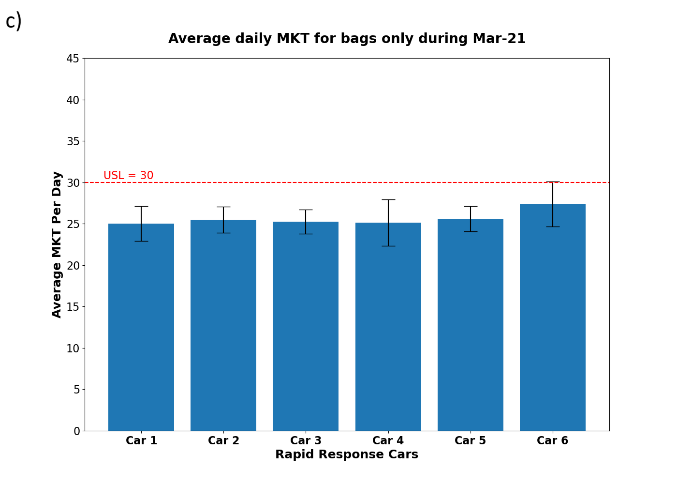

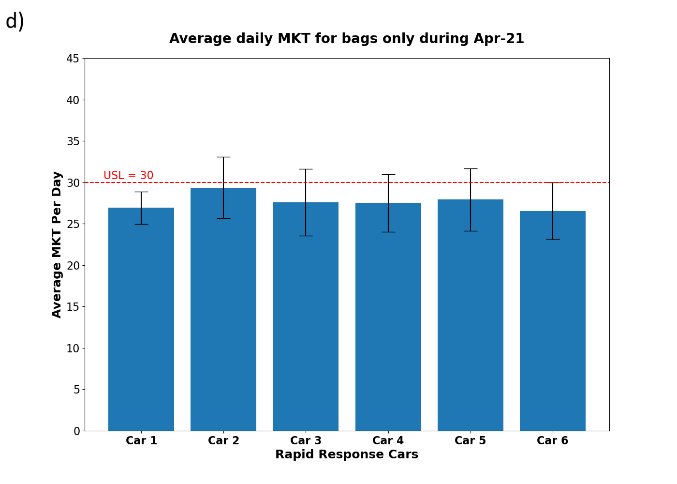

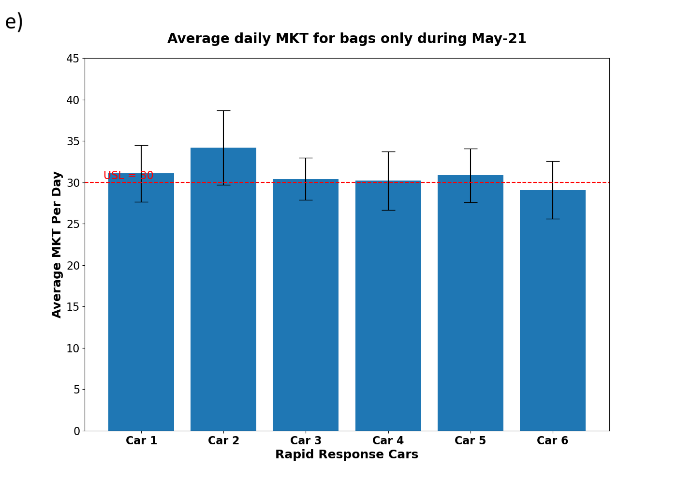

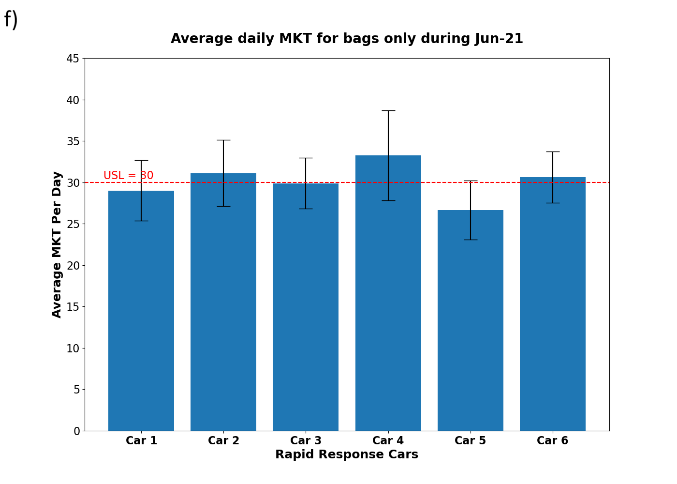

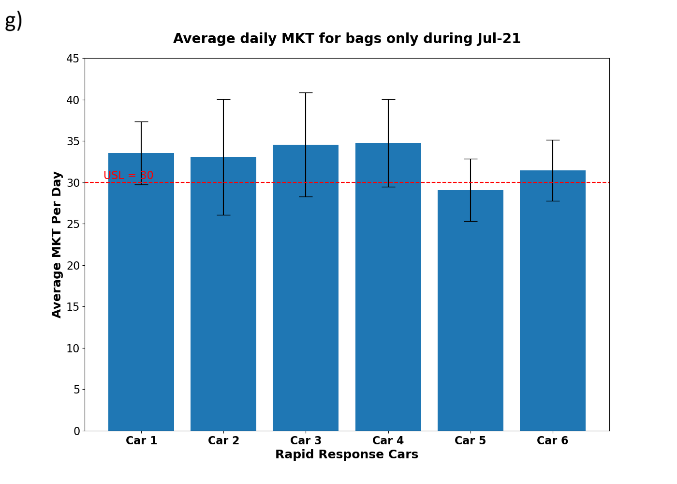

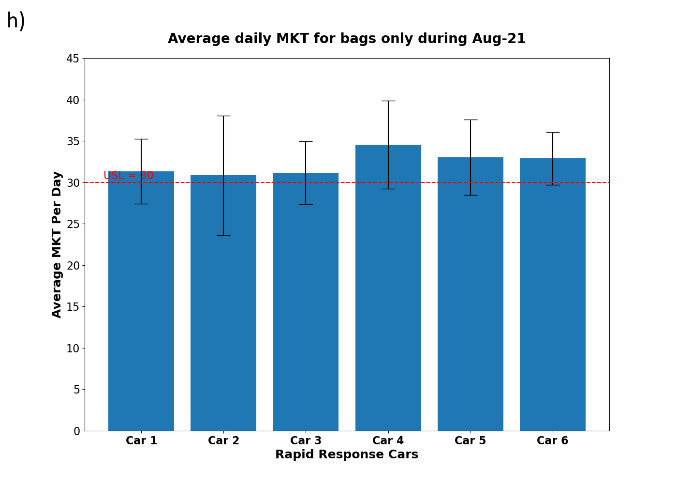

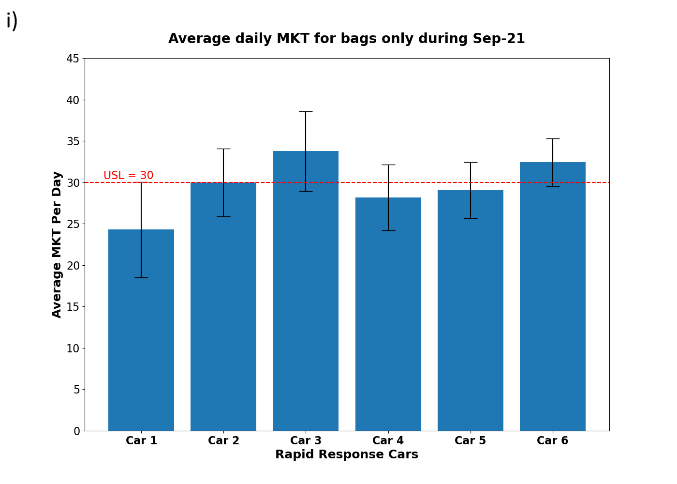

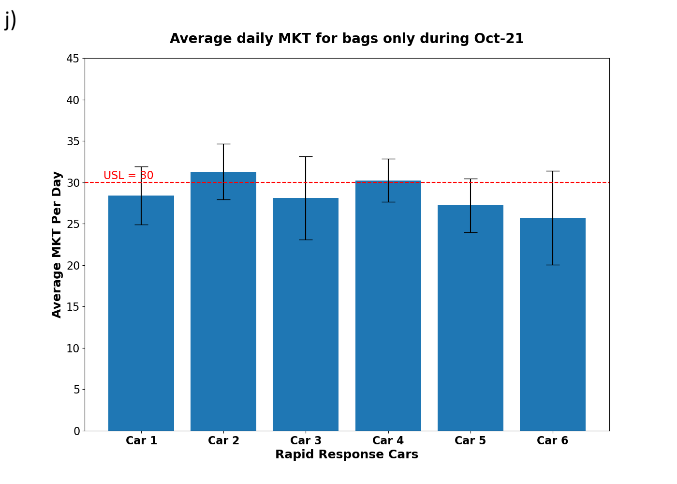

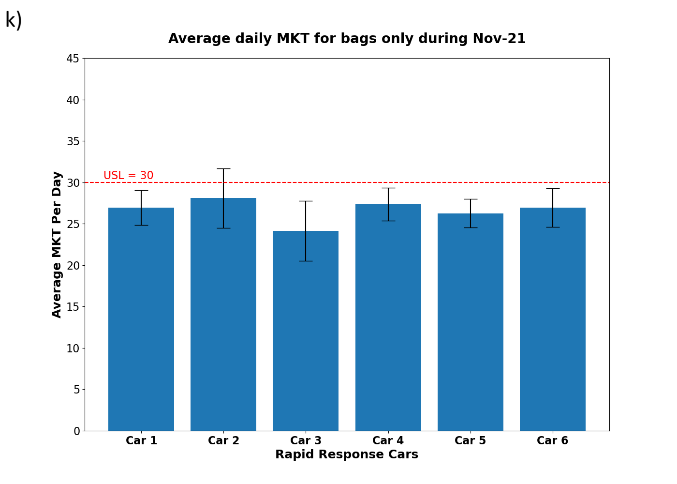

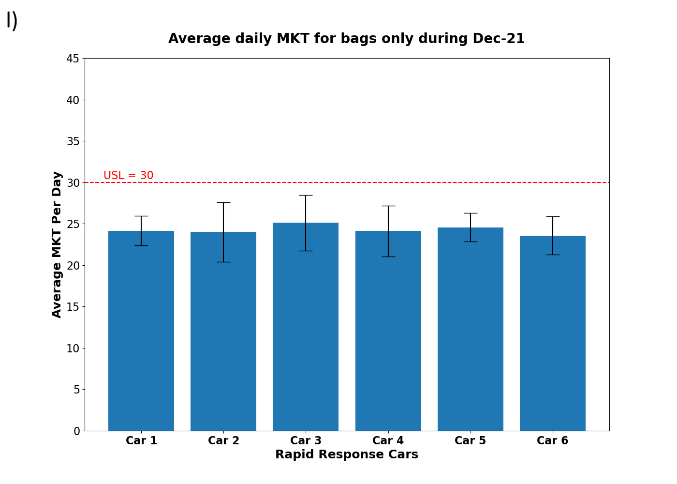

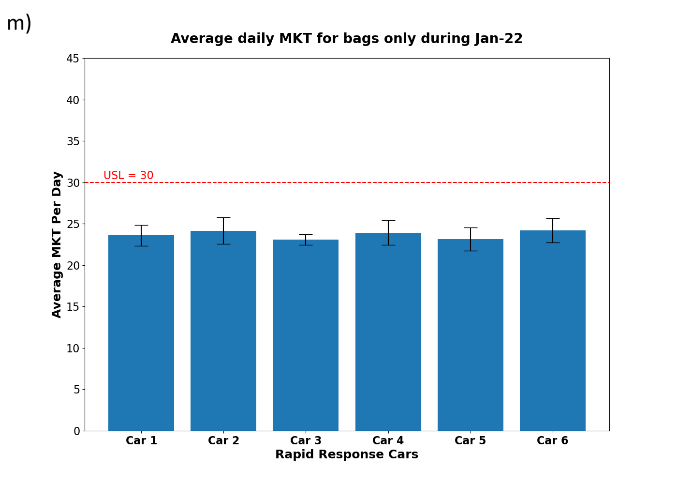
**

**Appendix 8: Average of daily mean kinetic temperature values reported over a month from inside the middle of rapid response cars only for each vehicle. Each month is presented separately in a graph (a-m). Dotted red line is the upper specification limit (USL) of 30 °C. Possible reasons leading to missing data have been identified as indicated in the inserted boxes.**

**
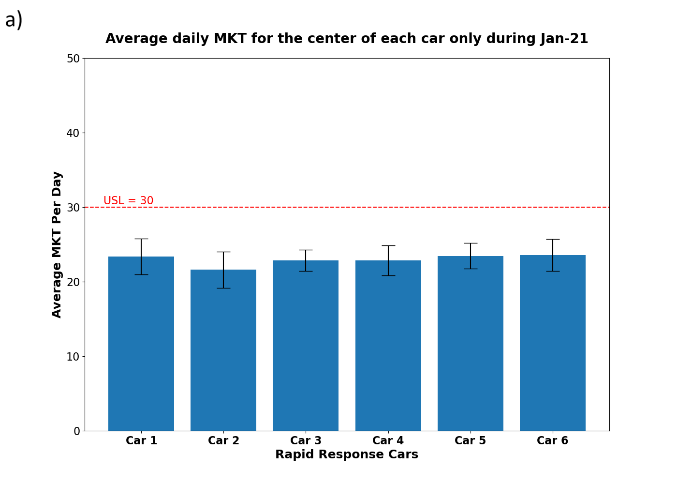

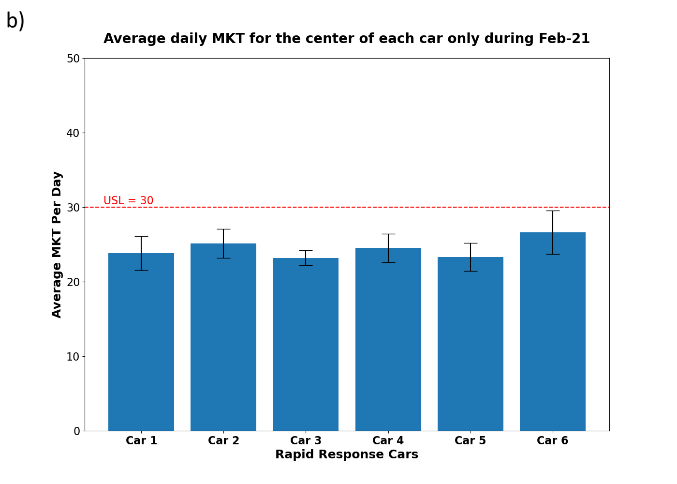

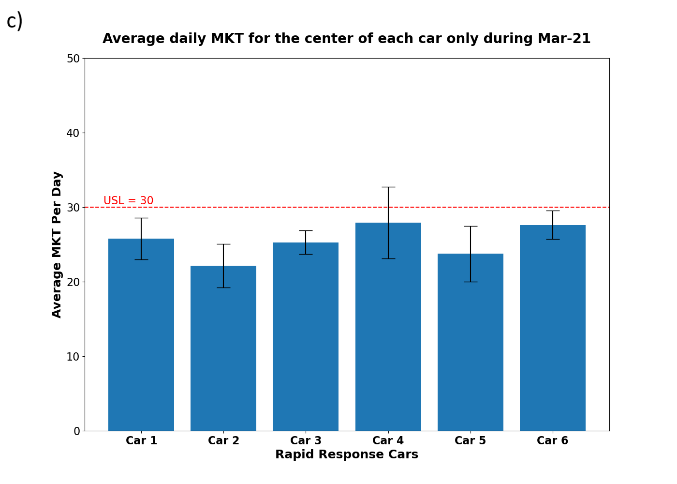

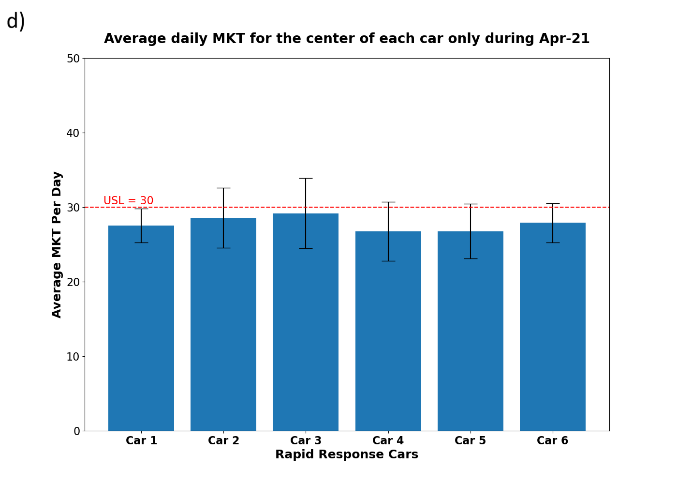

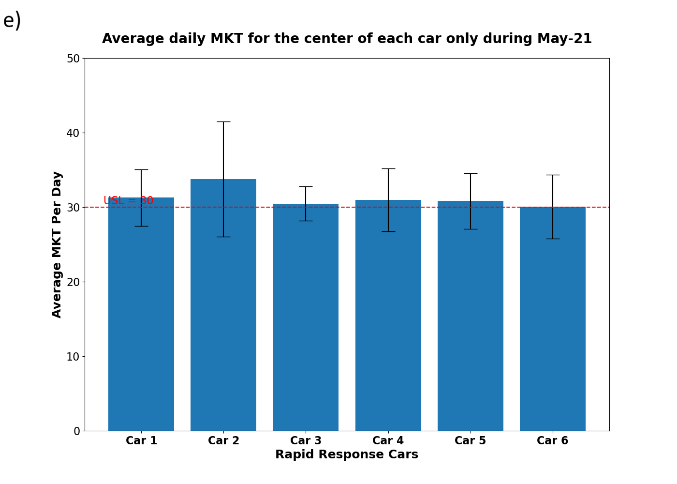

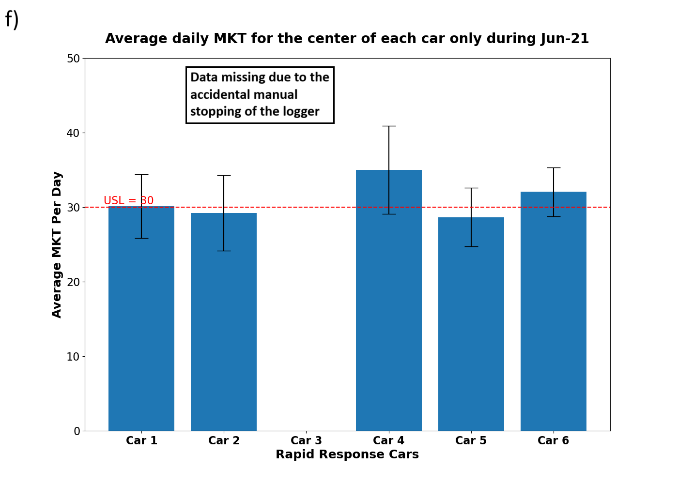

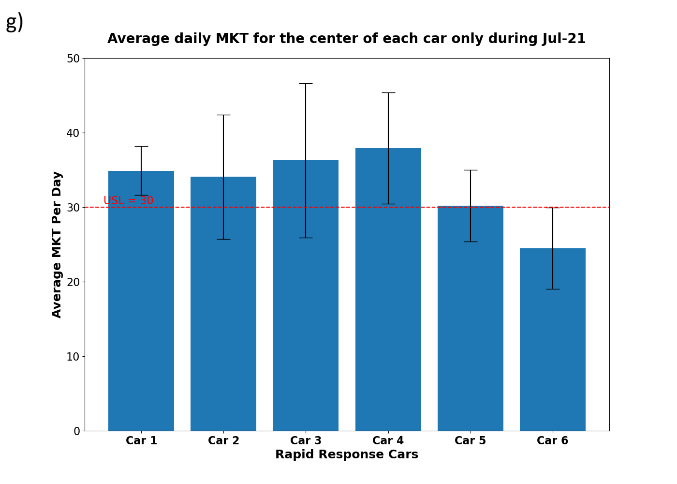

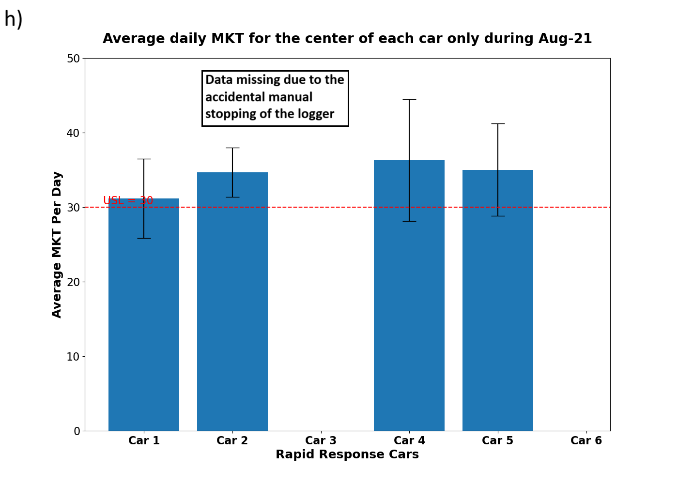

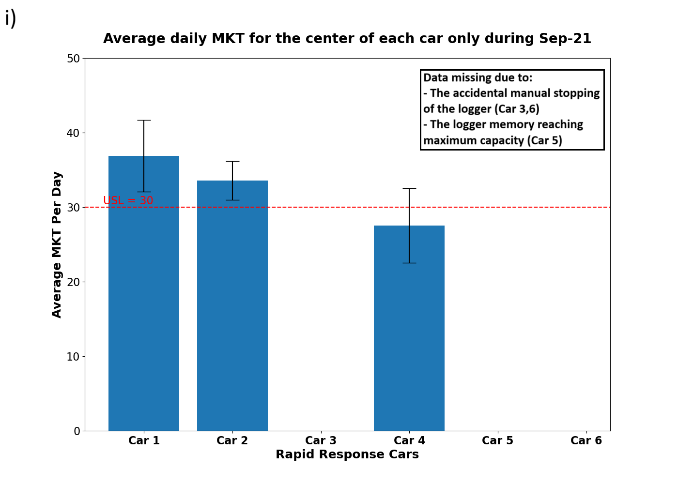

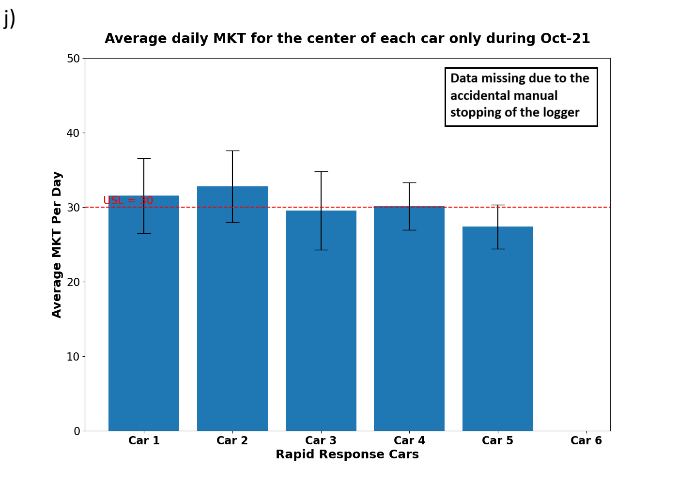

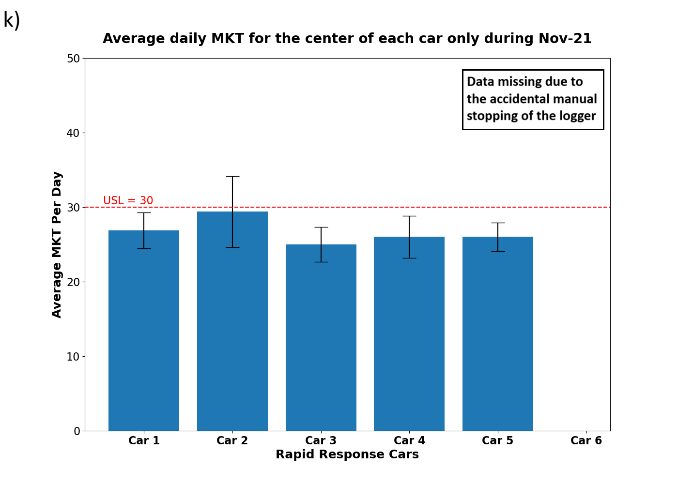

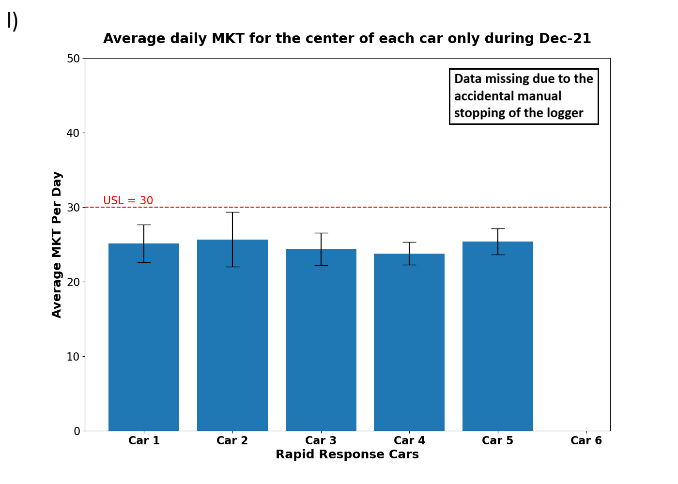

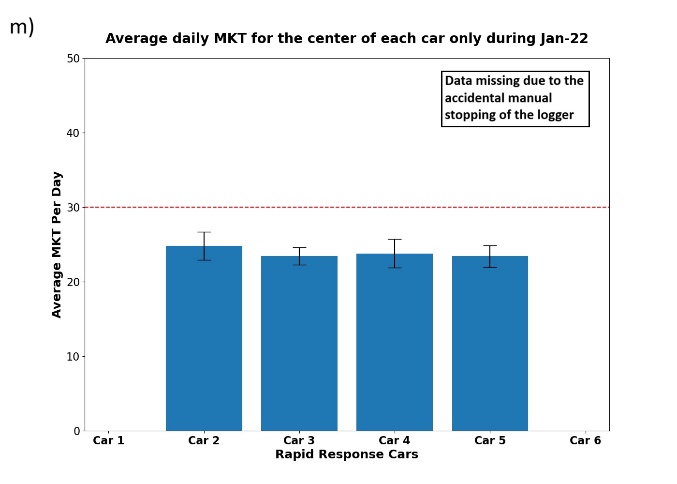
**
